# Truncated LKB1 nonenzymatically enhances Fas-induced apoptosis by acting as a surrogate of Smac

**DOI:** 10.1038/s41420-025-02570-1

**Published:** 2025-06-21

**Authors:** Yutaro Yamada, Mei Tsuchida, Takuya Noguchi, Takumi Yokosawa, Maki Mitsuya, Tatsuya Shimada, Daisuke Oikawa, Yusuke Hirata, Fuminori Tokunaga, Pascal Schneider, Atsushi Matsuzawa

**Affiliations:** 1 Laboratory of Health Chemistry, Graduate School of Pharmaceutical Sciences, Tohoku University, Sendai, Japan; 2 Department of Medical Biochemistry, School of Pharmacy, Iwate Medical University, Yahaba, Japan; 3 Department of Immunobiology, University of Lausanne, Epalinges, Switzerland; 4 Laboratory of Molecular Cell Biology, Institute for Molecular and Cellular Regulation (IMCR), Gunma University, Maebashi, Japan; 5 Department of Medical Biochemistry, Graduate School of Medicine, Osaka Metropolitan University, Osaka, Japan

**Keywords:** Apoptosis, Tumour-necrosis factors

## Abstract

Although liver kinase B1 (LKB1) has been established as a tumor suppressor kinase, its mechanism of action is incompletely understood. Here we describe a novel nonenzymatic function of LKB1 in cell death induced by Fas/CD95. In BID knockout HeLa cells, inactivation of mitochondrial outer membrane permeabilization (MOMP) prevents Smac-induced inhibition of X-linked inhibitor of apoptosis (XIAP), causing resistance to Fas-induced apoptosis. However, reexpression of LKB1 in those cells naturally deficient for endogenous LKB1 restored apoptosis. Mechanistically, caspase-8 activated by Fas processed LKB1 to a truncated form, tLKB1. Both WT and kinase-inactive LKB1 antagonized XIAP to restore apoptosis, but somatic mutants of LKB1 found in Peutz-Jeghers syndrome (PJS) failed to do so. Thus, in addition to the known caspase-8 / tBid / Smac / XIAP pro-apoptotic axis, our results unveil a novel one, caspase-8 / tLKB1 / XIAP that potentially contributes to the antitumor functions of LKB1.

## Introduction

Apoptosis is an evolutionarily conserved cellular suicide mechanism that removes unnecessary or harmful cells, and hence contributes to maintaining cellular homeostasis [1–3]. It is well established that apoptosis can be triggered by death receptors of the TNF receptor family, including Fas/CD95 and TNF-R1 [4–6]. Fas regulates homeostasis of lymphocytes and natural killer cells in the immune system, and confers susceptibility of target cells to cytotoxic effectors [7–9]. A deregulation of Fas activity is implicated in the pathogenesis of several diseases, including the autoimmune lymphoproliferative syndrome (ALPS) and cancer [10–12]. In response to Fas ligand (FasL), a multiprotein death-inducing signaling complex (DISC) containing FADD, and caspase-8 is recruited to the death domain-containing cytoplasmic tail of Fas [13–17]. On the DISC, the activation of caspase-8 is initiated, promoting processing of effector caspases. However, in most cell types, full activation of the effector caspases does not occur unless the intrinsic apoptotic pathway is engaged. The activation of the intrinsic pathway is triggered by caspase-8-dependent cleavage of BH3-interacting domain death agonist (BID) [18]. After the cleavage, the C-terminal part of truncated BID (tBID) translocates to mitochondria, and then initiates mitochondrial outer membrane permeabilization (MOMP) that allows mitochondrial release of pro-apoptotic factors such as cytochrome c, and second mitochondria-derived activator of caspases (Smac), leading to the activation of effector caspases including caspase-3 and caspase-7 [19, 20]. In particular, MOMP-dependent release of Smac into the cytoplasm unlocks the mechanism by which X-linked inhibitor of apoptosis protein (XIAP) strongly suppresses the intrinsic pathway by binding to the activated forms of caspase-3 and caspase-7. Interestingly, deletion of XIAP sensitizes cells to death receptor-induced apoptosis even in the absence of MOMP, suggesting that XIAP is an essential inhibitor of the intrinsic pathway [21–23].

Liver kinase B1 (LKB1), also called serine/threonine kinase 11 (STK11), is a ubiquitously expressed serine/threonine kinase originally identified as the tumor-suppressor gene responsible for an inherited cancer disorder, Peutz-Jeghers syndrome (PJS), a rare autosomal dominant intestinal hamartomatous polyposis syndrome associated with perioral melanotic pigmentations and increased risk of cancer, including pancreatic, breast, lung, ovary, and uterine carcinomas [24–26]. Loss of function LKB1 mutations are also found in several sporadic cancers, especially in colorectal, pancreatic, cervical, non-small cell lung carcinoma and melanoma, which seems to be closely associated with accelerated disease progression and poor clinical prognosis [27–29]. Functionally, LKB1 contributes to the regulation of intracellular energy levels, cell proliferation, autophagy, apoptosis, and cell polarity by activating its downstream kinases, including mainly AMP-activated protein kinase (AMPK) families [27, 30–32]. Among them, suppression of mammalian target of rapamycin (mTOR) signaling pathway by the LKB1-AMPK pathway is considered to be an important tumor-suppressive function [33]. Moreover, LKB1 phosphorylates tumor suppressor p53 at serine (Ser)-392 upon DNA damage, leading to the stabilization and activation of p53 [34, 35]. On the other hand, LKB1 controls cell polarity independently of its kinase activity [36–38]. Since LKB1-dependent regulation of cell polarity is highly conserved from C. elegans to mammals, it is possible that the kinase activity-independent functions of LKB1 play critical roles in various cellular processes.

However, tumor-suppressive function of LKB1 exerted independently of its kinase activity has not been reported. On the other hand, previous studies have reported that overexpression or reconstitution of LKB1 in cancer cells promotes apoptosis, although the underlying mechanisms remain unclear [39–41]. Notably, recent evidence indicates that LKB1 mutations may allow tumor cells to evade cytotoxic responses mediated by natural killer cells [42, 43]. Given that NK cells induce tumor cell death in part through FasL-induced apoptosis, these findings raise the possibility that tumor-suppressive function of LKB1 is exerted by promoting FasL-induced apoptosis. Thus, these observations prompted us to investigate a potential mechanistic link between LKB1 and FasL-induced apoptosis.

In this study, we found that LKB1 can stimulate Fas-mediated apoptosis independently of BID-mediated MOMP. Upon Fas stimulation, LKB1 is truncated by caspase-8, and then the truncated LKB1 (tLKB1) promotes the proteasomal degradation of XIAP. Considering that the proteasomal degradation of XIAP is promoted by cytosolic Smac released by BID-mediated MOMP, tLKB1 may act as a surrogate of Smac when the BID-mediated MOMP is disordered. Thus, tLKB1 serves as a bypass mechanism to ensure the induction of cell death, which provides novel insight into the antitumor functions of LKB1.

## Results

### LKB1 nonenzymatically promotes Fas-induced apoptosis in BID KO HeLa cells

**Fig. 1:**
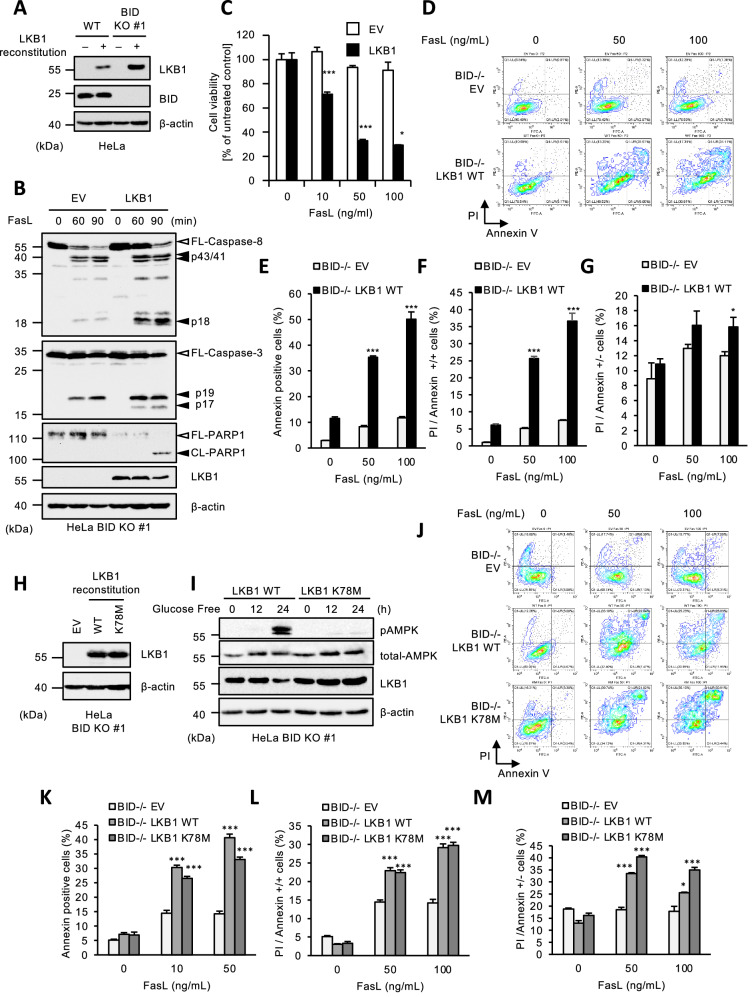
LKB1 nonenzymatically promotes Fas-induced apoptosis in BID KO HeLa cells. **A** Immunoblot analysis of LKB1 in LKB1 reconstituted HeLa WT or BID KO cells. Cell lysates were subjected to immunoblotting with the indicated antibodies. β-actin was used as a loading control. **B** Empty vector (EV) or LKB1 reconstituted BID KO HeLa cells were treated with Fc-FasL (100 ng/mL) for indicated periods, and then cell lysates were subjected to immunoblotting with the indicated antibodies. **C** Empty vector (EV) or LKB1 reconstituted BID KO HeLa cells were treated with Fc-FasL (0,10,50,100 ng/mL) for 12 h. Cell viability was determined by PMS/MTS assay. Data shown are the mean ± SD (n = 3). Statistical significance was tested using an unpaired Student’s t test; *p < 0.05, ***p < 0.001, (vs. control cells). **D** Empty vector (EV) or LKB1 reconstituted BID KO HeLa cells were treated with Fc-FasL (0, 50,100 ng/mL) for 12 h. Apoptotic cells were labeled with annexin V-FITC and PI for 15 min, and analyzed by FACS. The data is presented as FITC-PE fluorescence density plots. **E** Quantification of the percentage of Annexin V-positive cells shown in Fig. 1D. Data shown are the mean ± SD (n = 3). Statistical significance was tested using an unpaired Student’s t test; ***p < 0.001, (vs. control cells). **F** Quantification of the percentage of PI and annexin V double-positive cells shown in Fig. 1D. Data shown are the mean ± SD (n = 3). Statistical significance was tested using an unpaired Student’s t test; ***p < 0.001, (vs. control cells). **G** Quantification of the percentage of PI-positive/Annexin V-negative cells shown in Fig. 1D. Data shown are the mean ± SD (n = 3). Statistical significance was tested using an unpaired Student’s t test; *p < 0.05, (vs. control cells). **H** Immunoblot analysis of LKB1 in LKB1 WT or K78M reconstituted HeLa BID KO cells. Cell lysates were subjected to immunoblotting with the indicated antibodies. **I** LKB1 WT or K78M reconstituted BID KO HeLa cells were cultured with glucose free medium for 0, 12 or 24 h, and then cell lysates were subjected to immunoblotting with the indicated antibodies. **J** Empty vector (EV) or LKB1 (WT or K78M) reconstituted BID KO HeLa cells were treated with Fc-FasL (0, 50,100 ng/mL) for 12 h. Apoptotic cells were labeled with annexin V-FITC and PI for 15 min, and analyzed by FACS. Data is presented as FITC-PE fluorescence density plots. **K** Quantification of the percentage of annexin V-positive cells shown in Fig. 1J. Data shown are the mean ± SD (n = 3). Statistical significance was tested using an unpaired Student’s t test; ***p < 0.001, (vs. control cells). **L** Quantification of the percentage of PI and annexin V double-positive cells shown in Fig. 1J. Data shown are the mean ± SD (n = 3). Statistical significance was tested using an unpaired Student’s t test; ***p < 0.001, (vs. control cells). **M** Quantification of the percentage of PI-positive/Annexin V-negative cells shown in Fig. 1J. Data shown are the mean ± SD (n = 3). Statistical significance was tested using an unpaired Student’s t test; ***p < 0.001, (vs. control cells). All data are representative of at least three biologically independent replicates.

It is well known that BID is essential for the induction of Fas-induced apoptosis, because tBID produced by caspase-8-dependent cleavage is required to induce Bax/Bak-mediated MOMP. Indeed, JC-1 assay to detect loss of mitochondrial membrane potential (MMP) revealed that soluble Fas ligand (Fc-FasL), purified as previously described [44], fails to induce MOMP in BID KO HeLa cells (Supplemental Fig. 1A). Consistent with this finding, BID KO HeLa cells were strongly resistant to Fas-induced apoptosis (Supplemental Figs. 1B–E). Moreover, we observed that the processing of caspase-3 p19 fragment to p17 fragment that is required for the full activation of caspase-3 is completely suppressed in BID KO HeLa cells, because the caspase-3 processing is mediated by Smac released from mitochondria in a MOMP-dependent manner (Supplemental Fig. 1F). Unexpectedly, we found that reconstitution of LKB1 in HeLa cells that harbor a homozygous deletion of the LKB1 locus restored the caspase-3 processing, and caspase-dependent cleavage of PARP-1 even in the absence of BID (Fig. 1A, B) [45]. The reconstruction of LKB1 did not affect the caspase-8 processing to p43 fragment, but interestingly, promoted the processing to p18 fragment (Fig. 1B). In this regard, it has been reported that active caspase-3 promotes the processing of caspase-8 from p43 to p18 fragment [46], and indeed, the processing of caspase-8 to p18 fragment was suppressed in caspase-3-deficient cells that we have established previously (Supplemental Fig. 2A) [47]. These results suggested that LKB1 does not affect the caspase-8 processing to p43 fragment mediated by the DISC, but promotes the caspase-8 processing to p18 fragment mediated by the caspase-3 activation (Fig. 1B). In addition, reconstitution of LKB1 in BID KO HeLa cells exhibited increased susceptibility to Fas-induced apoptosis when compared with control cells that do not express LKB1 (Fig. 1C). The apoptosis-promoting effects of LKB1 was observed in BID expressing cells, but its effect was slight (Supplemental Figs. 3A, B). Therefore, LKB1 seems to be important for inducing apoptosis in MOMP-deficient cells. FACS analysis revealed that annexin V (+) positive (early phase apoptosis) cells and, annexin V ( + )/PI (+) (late phase apoptosis) cells were significantly increased in LKB1 reconstituted HeLa cells, suggesting that LKB1 promotes Fas-induced apoptosis independently of BID-dependent mechanisms (Fig. 1D–G). We therefore explored the mechanisms by which LKB1 promotes Fas-induced apoptosis in BID KO HeLa cells. First, to examine whether kinase activity of LKB1 is required for LKB1-mediated apoptosis, we reconstituted LKB1 K78M, the kinase-dead form of LKB1 mutant (mutation of Lys 78 to Met), in BID KO HeLa cells (Fig. 1H), and confirmed that LKB1 K78M mutant fails to activate AMPKα due to loss of the kinase activity (Fig. 1I). However, interestingly, LKB1 K78M reconstituted BID KO HeLa cells exhibited increased susceptibility to Fas-induced apoptosis to the same extent as LKB1 WT reconstituted BID KO HeLa cells (Fig. 1J–M). Therefore, these results suggest that LKB1 promotes Fas-induced apoptosis in BID KO HeLa cells in a manner independent from kinase activity.

### LKB1 promotes Fas-induced apoptosis through the accelerated self-degradation of XIAP in BID KO HeLa cells

**Fig. 2:**
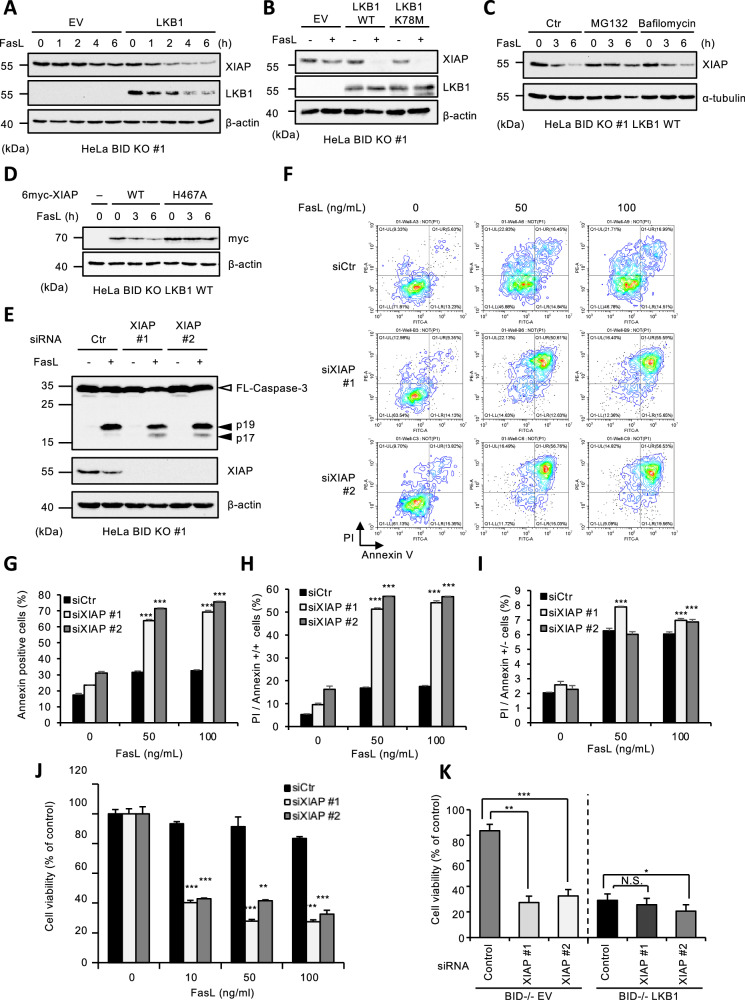
LKB1 promotes Fas-induced apoptosis through the accelerated self-degradation of XIAP in BID KO HeLa cells. **A** Empty vector (EV) or LKB1 reconstituted BID KO HeLa cells were treated with Fc-FasL (100 ng/mL) for 0, 1, 2, 4 or 6 h, and then cell lysates were subjected to immunoblotting with the indicated antibodies. **B** Empty vector (EV) or LKB1 (WT or K78M) reconstituted BID KO HeLa cells were treated with Fc-FasL (100 ng/mL) for 4 h, and then cell lysates were subjected to immunoblotting with the indicated antibodies. **C** LKB1 reconstituted BID KO HeLa cells were treated with Fc-FasL (100 ng/mL), and MG132 (10 μM) or bafilomycin (5 nM) for 0, 3 or 6 h, and then cell lysates were subjected to immunoblotting with the indicated antibodies. **D** LKB1 reconstituted BID KO HeLa cells were transfected with 6myc-XIAP (WT or H467A) for 24 h, and then treated with Fc-FasL (100 ng/mL) for 0, 3 or 6 h. Cell lysates were subjected to immunoblotting with the indicated antibodies. **E** BID KO HeLa cells were transfected with siRNA for negative control (siCtr) or XIAP (siXIAP #1 or siXIAP #2) for 24 h, and then treated with Fc-FasL (100 ng/mL) for 2 h. Cell lysates were subjected to immunoblotting with the indicated antibodies. **F** BID KO HeLa cells were transfected with siRNA for negative control (siCtr) or XIAP (siXIAP #1 or siXIAP #2) for 24 h, and then treated with Fc-FasL (0, 50, 100 ng/mL) for 12 h. Apoptotic cells were labeled with annexin V-FITC and PI for 15 min, and analyzed by FACS. Data is presented as FITC-PE fluorescence density plots. **G** Quantification of the percentage of annexin V-positive cells shown in Fig. 2F. Data shown are the mean ± SD (n = 3). Statistical significance was tested using an unpaired Student’s t test; ***p < 0.001, (vs. control cells). **H** Quantification of the percentage of PI and annexin V double-positive cells shown in Fig. 2F. Data shown are the mean ± SD (n = 3). Statistical significance was tested using an unpaired Student’s t test; ***p < 0.001, (vs. control cells). **I** Quantification of the percentage of PI-positive/Annexin V-negative cells shown in Fig. 2F. Data shown are the mean ± SD (n = 3). Statistical significance was tested using an unpaired Student’s t test; ***p < 0.001, (vs. control cells). **J** BID KO HeLa cells were transfected with siRNA for negative control (siCtr) or XIAP (siXIAP #1 or siXIAP #2) for 24 h, and then treated with Fc-FasL (0, 10, 50, 100 ng/mL) for 12 h. Cell viability was determined by PMS/MTS assay. Data shown are the mean ± SD (n = 3). Statistical significance was tested using an unpaired Student’s t test; **p < 0.01, ***p < 0.001, (vs. control cells). **K** Empty vector (EV) or LKB1 reconstituted BID KO HeLa cells were transfected with siRNA for negative control (Control) or XIAP (siXIAP #1 or siXIAP #2) for 24 h, and then treated with Fc-FasL (100 ng/mL) for 12 h. Cell viability was determined by PMS/MTS assay. Data shown are the mean ± SD (n = 3). Statistical significance was tested using an unpaired Student’s t test; *p < 0.05, ***p < 0.001, (vs. control cells). All data are representative of at least three biologically independent replicates.

An important role of MOMP is to promote the degradation of XIAP. We therefore speculated that LKB1 may affect the XIAP degradation. As shown in Fig. 2A, XIAP degradation in BID KO HeLa cells was promoted in the presence of LKB1 WT. This was also true for cells reconstituted with the kinase inactive LKB1 K78M (Fig. 2B). The degradation of XIAP in LKB1 reconstituted BID KO HeLa cells was inhibited by the cotreatment with the proteasome inhibitor MG132 but not the autophagy inhibitor bafilomycin A1, and XIAP H467A mutant that lost E3 Ub ligase function exhibited resistant to degradation (Fig. 2C, D). Collectively, these data suggest that LKB1 somehow links Fas activation to XIAP self-degradation in BID KO HeLa cells. To examine whether the promoted degradation of XIAP induced by LKB1 sensitizes BID KO HeLa cells to apoptosis, we performed knockdown experiments using small interfering RNA (siRNA) against XIAP. As expected, the XIAP knockdown restored processing of caspase-3 to the p17 fragment (Fig. 2E). Consistent with caspase-3 processing, both FACS analysis (Fig. 2F–I) and MTS assay (Fig. 2J) revealed that the XIAP knockdown sensitized BID KO HeLa cells to Fas-induced apoptosis. Moreover, the effect of XIAP knockdown on apoptosis was much smaller in LKB1 WT reconstituted BID KO HeLa cells when compared with control cells that do not express LKB1 (Fig. 2K). Therefore, these findings suggest that LKB1 promotes Fas-induced apoptosis through the accelerated self-degradation of XIAP in BID KO HeLa cells.

### Truncated LKB1 leads to the degradation of XIAP in BID KO HeLa cells

**Fig. 3:**
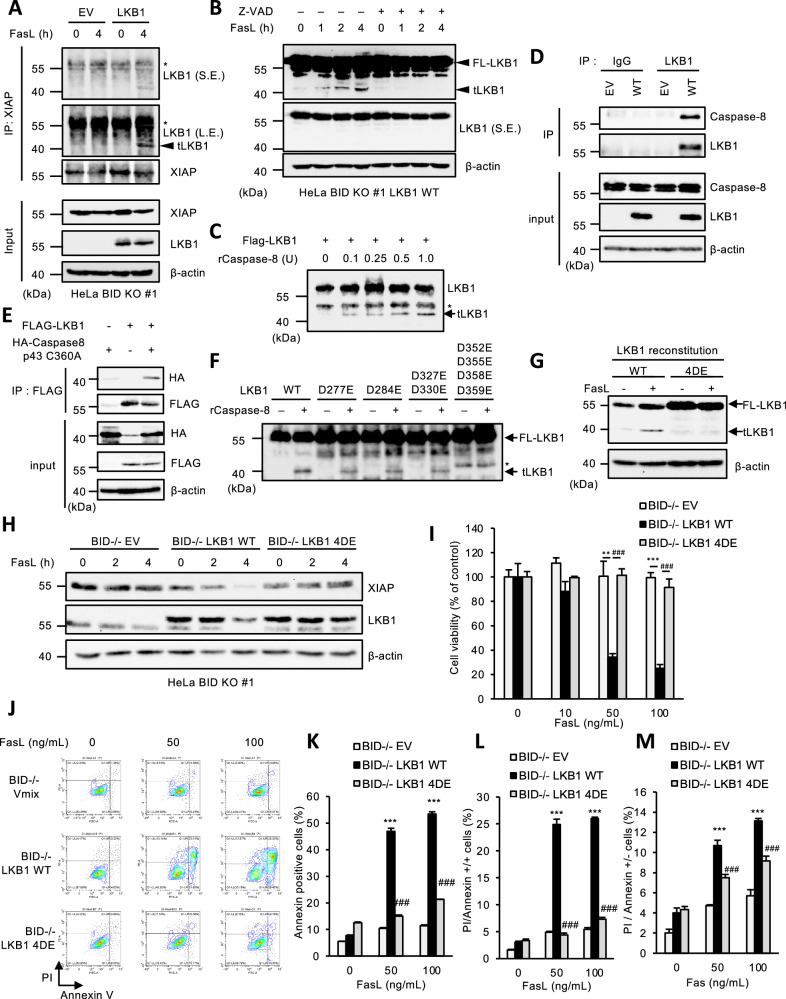
Truncated LKB1 leads to the degradation of XIAP in BID KO HeLa cells. **A** Empty vector (EV) or LKB1 reconstituted BID KO HeLa cells were treated with Fc-FasL (100 ng/mL) for 4 h, then immunoprecipitated with the anti-XIAP antibody, and subjected to immunoblotting with the indicated antibodies. The band indicated by an asterisk is a non-specific band that does not change upon LKB1 expression. The arrowhead indicates tLKB1. S.E.: short exposure. L.E.: long exposure. **B** LKB1 reconstituted BID KO HeLa cells were treated with Fc-FasL (100 ng/mL) and z-VAD (20 μM) for 0, 1, 2 or 4 h, and then cell lysates were subjected to immunoblotting with the indicated antibodies. **C** FLAG-LKB1, affinity-purified from 293 A cells overexpressing FLAG-LKB1, was reacted with recombinant active caspase-8 (0, 0.1, 0.25, 0.5, 1.0 units), and subjected to immunoblotting with the indicated antibodies. The band indicated with an asterisk is a non-specific band that is not affected by recombinant caspase-8. **D** Cell lysates from empty vector (EV) or LKB1-reconstituted BID KO HeLa cells were immunoprecipitated with protein G-Sepharose beads using the indicated antibodies, and subjected to immunoblotting with the indicated antibodies. **E** HEK293A cells were transfected with FLAG-LKB1 and/or HA-Caspase-8 p43 (C360A) plasmid for 24 h, and immunoprecipitated anti-FLAG-tagged agarose beads, and subjected to immunoblotting with the indicated antibodies. **F** FLAG-LKB1 (WT, D277E, D284E, D327/330E, D352/355/358/359E), affinity-purified from 293 A cells overexpressing FLAG-LKB1, were reacted with recombinant active caspase-8 (0.5 units), and subjected to immunoblotting with the indicated antibodies. The band indicated by an asterisk is a non-specific band that is not affected by recombinant caspase-8. **G** FLAG-LKB1 (WT or 4DE) reconstituted BID KO HeLa cells were treated with Fc-FasL (100 ng/mL) for 4 h, and then cell lysates were subjected to immunoblotting with the indicated antibodies. **H** Empty vector (EV) or LKB1 (WT or 4DE) reconstituted BID KO HeLa cells were treated with Fc-FasL (100 ng/mL) for 0, 2 or 4 h, and then cell lysates were subjected to immunoblotting with the indicated antibodies. **I** Empty vector (EV) or LKB1 (WT or 4DE) reconstituted BID KO HeLa cells were treated with Fc-FasL (0, 10, 50, 100 ng/mL) for 12 h. Cell viability was determined by PMS/MTS assay. Data shown are the mean ± SD (n = 3). Statistical significance was tested using an unpaired Student’s t test; ***p < 0.001, (vs. EV cells), ###*p* < 0.001 (vs. LKB1 WT cells). **J** Empty vector (EV) or LKB1 (WT/4DE) reconstituted BID KO HeLa cells were treated with Fc-FasL (0, 50, 100 ng/mL) for 12 h. Apoptotic cells were labeled with annexin V-FITC and PI for 15 min, and analyzed by FACS. Data is presented as FITC-PE fluorescence density plots. **K** Quantification of the percentage of Annexin V-positive cells shown in Fig. 3J. Data shown are the mean ± SD (n = 3). Statistical significance was tested using an unpaired Student’s t test; ***p < 0.001, (vs. EV cells), ###*p* < 0.001 (vs. LKB1 WT cells). **L** Quantification of the percentage of PI and annexin V double-positive cells shown in Fig. 3J. Data shown are the mean ± SD (n = 3). Statistical significance was tested using an unpaired Student’s t test; ***p < 0.001, (vs. EV cells), ###*p* < 0.001 (vs. LKB1 WT cells). **M** Quantification of the percentage of PI-positive/Annexin V-negative cells shown in Fig. 3J. Data shown are the mean ± SD (n = 3). Statistical significance was tested using an unpaired Student’s t test; ***p < 0.001, (vs. EV cells), ###*p* < 0.001 (vs. LKB1 WT cells). All data are representative of at least three biologically independent replicates.

We next examined how LKB1 promotes the self-degradation of XIAP in BID KO HeLa cells. First, we tested the interaction between LKB1 and XIAP. Interestingly, an immunoprecipitation analysis revealed that immunoprecipitated LKB1 with XIAP was smaller (40 kDa) than expected (55 kDa) (Fig. 3A). We therefore speculated that a truncated form of LKB1 binds to XIAP, and then investigated how LKB1 gets truncated. Interestingly, we found that LKB1 is truncated in a FasL-dependent manner, although the amount of truncated LKB1 (tLKB1) is only small (Fig. 3B). Moreover, the truncation of LKB1 was inhibited by the pan-caspase inhibitor z-VAD-fmk (Fig. 3B). Indeed, in vitro truncation assay showed that recombinant active caspase-8 (rcaspase-8) predominantly cleaved LKB1 as compared with rcaspase-3 and rcaspase-9 (Fig. 3C and Supplemental Fig. 4A-E). Although how its selectivity is determined is unclear, we observed that LKB1 clearly binds to both endogenous caspase-8 (Fig. 3D) and p43 fragment (Fig. 3E). Therefore, caspase-8 is likely to cleave LKB1 as a substrate physiologically. We next defined the caspase-8 cleavage sites in LKB1. LKB1 possesses several aspartic acids (D) that can be cleaved by caspase-8 (Fig. 3F). Considering that the recognition site of the anti-LKB1 monoclonal antibody used to detect 40 kDa LKB1 is at the N-terminal side, we focused on the aspartic acid residues at the C-terminal side, which are common between human and mouse, and substituted them for glutamic acids that are not recognized by caspase-8. As shown in Fig. 3F, only LKB1 D352/355/358/359E (4DE) mutant in which aspartic acids 352, 355, 358, and 359 were substituted by glutamic acids (E) was not truncated to 40 kDa even in the presence of recombinant activated caspase-8. Additionally, LKB1 4DE mutant reconstituted in BID KO cells was not processed during FasL treatment, and failed to promote XIAP degradation, suggesting that LKB1 is truncated at D352, D355, D358, or D359 by caspase-8 in Fas-dependent manner (Fig. 3G, H). Moreover, LKB1 4DE mutant-reconstituted BID KO cells exhibited resistance to Fas-induced apoptosis, when analyzing both MTS assay and FACS analysis (Fig. 3I–M). On the contrary, transient expression of truncated LKB1, which lacks the region after D352 of LKB1, sufficiently reduced the expression of XIAP, and promoted the activation of caspase-3 (Supplemental Figs. 5A, B). In addition, transient overexpression of tLKB1 was sufficient to induce cell death in BID-deficient HeLa cells (Supplemental Figs. 5C–F). Collectively, these observations suggest that LKB1 truncated by caspase-8 is required and sufficient for XIAP degradation and Fas-induced apoptosis in BID KO cells.

### A four amino acid sequence of LKB1 resembling an IAP binding motif is required for Fas-induced apoptosis in BID KO HeLa cells

**Fig. 4:**
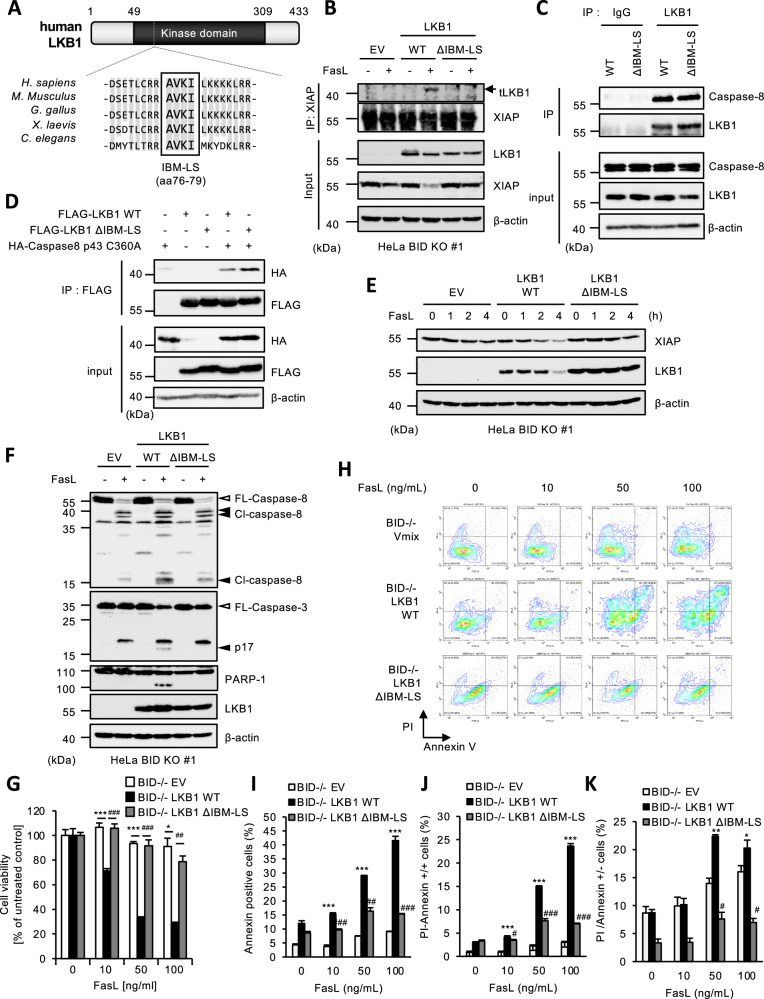
A four amino acid sequence of LKB1 resembling an IAP binding motif is required for Fas-induced apoptosis in BID KO HeLa cells. **A** Alignment of an IAP binding motif-like sequence (IBM-LS) of LKB1 for the indicated species. aa76-79 of human LKB1 correspond to the IBM-LS. **B** Empty vector (EV) or LKB1 (WT or ΔIBM-LS) reconstituted BID KO HeLa cells were treated with Fc-FasL (100 ng/mL) for 4 h, then immunoprecipitated with the anti-XIAP antibody, and subjected to immunoblotting with the indicated antibodies. **C** Cell lysates from LKB1 (WT/ΔIBM-LS) reconstituted BID KO HeLa cells were immunoprecipitated with protein G-Sepharose beads using the indicated antibodies, and subjected to immunoblotting with the indicated antibodies. **D** HEK293A cells were transfected with FLAG-LKB1 (WT/ΔIBM-LS) and/or HA-Caspase-8 p43 (C360A) plasmid for 24 h, and immunoprecipitated anti-FLAG-tagged agarose beads, and subjected to immunoblotting with the indicated antibodies. **E** Empty vector (EV) or LKB1 (WT or ΔIBM-LS) reconstituted BID KO HeLa cells were treated with Fc-FasL (100 ng/mL) for 0, 1, 2 or 4 h, and then cell lysates were subjected to immunoblotting with the indicated antibodies. **F** Empty vector (EV) or LKB1 (WT or ΔIBM-LD) reconstructed BID KO HeLa cells were treated with Fc-FasL (100 ng/mL) for 3 h, and then the cell lysates were subjected to immunoblotting with the indicated antibodies. **G** Empty vector (EV) or LKB1 (WT or ΔIBM-LS) reconstituted BID KO HeLa cells were treated with Fc-FasL (0, 10, 50, 100 ng/mL) for 12 h. Cell viability was determined by PMS/MTS assay. Data shown are the mean ± SD (n = 3). Statistical significance was tested using an unpaired Student’s t test; *p < 0.05, ***p < 0.001, (vs. EV cells), ##*p* < 0.01, ###*p* < 0.001 (vs. LKB1 WT cells) (**H**) Empty vector (EV) or LKB1 (WT or ΔIBM-LS) reconstituted BID KO HeLa cells were treated with Fc-FasL (0, 10, 50, 100 ng/mL) for 12 h. Apoptotic cells were labeled with annexin V-FITC and PI for 15 min, and analyzed by FACS. Data is presented as FITC-PE fluorescence density plots. **I** Quantification of the percentage of annexin V-positive cells shown in Fig. 4H. Data shown are the mean ± SD (n = 3). Statistical significance was tested using an unpaired Student’s t test; ***p < 0.001, (vs. EV cells), ###*p* < 0.001 (vs. LKB1 WT cells). **J** Quantification of the percentage of PI and annexin V double-positive cells shown in Fig. 4H. Data shown are the mean ± SD (n = 3). Statistical significance was tested using an unpaired Student’s t test; ***p < 0.001, (vs. EV cells), ###*p* < 0.001 (vs. LKB1 WT cells). **K** Quantification of the percentage of PI-positive/Annexin V-negative cells shown in Fig. 4H. Data shown are the mean ± SD (n = 3). Statistical significance was tested using an unpaired Student’s t test; ***p < 0.001, (vs. EV cells), ###*p* < 0.001 (vs. LKB1 WT cells). All data are representative of at least three biologically independent replicates.

The XIAP-binding proteins, such as Smac, HtrA2/Omi, and caspase-9, bind to the BIR domain of XIAP through a four-amino acid motif sequence called IAP binding motif (IBM) exposed at the N-terminus [48, 49]. Interestingly, a sequence similar to IBM was found in amino acid residues 76 to 79 within the kinase domain of human LKB1 (Fig. 4A). However, unlike other IBM-containing proteins, the IBM-like sequence (IBM-LS) in LKB1 is not positioned at the N-terminus. Therefore, although it was unclear whether IBM-LS functions as an IBM, we investigated the involvement of IBM-LS in the LKB1-dependent XIAP degradation. As shown in Fig. 4B–D, LKB1 mutant lacking IBM-LS (LKB1 ΔIBM-LS) bound to caspase-8, but failed to bind to XIAP. Moreover, LKB1 ΔIBM-LS failed to promote XIAP degradation (Fig. 4E), and the processing of caspase-3 p19 fragment to p17 fragment (Fig. 4F). Consistent with these observations, BID KO HeLa cells reconstituted with LKB1 ΔIBM-LS were resistant to Fas-induced apoptosis, as witnessed in both MTS assay and FACS analysis (Fig. 4G–K). Collectively, these observations suggest that binding of LKB1 with XIAP through IBM-LS in LKB1 is required for Fas-induced apoptosis following the XIAP degradation in BID KO HeLa cells.

### Several LKB1 mutants found in PJS fail to promote Fas-induced apoptosis in BID KO HeLa cells

**Fig. 5:**
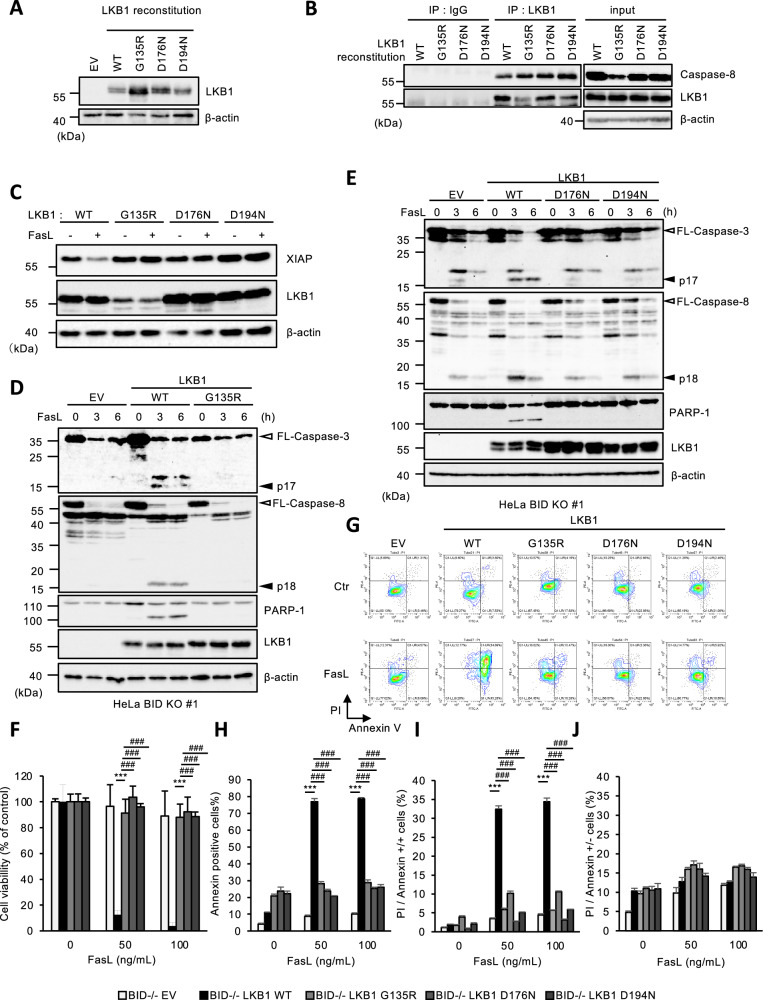
Several LKB1 mutants found in PJS failed to promote Fas-induced apoptosis in BID KO HeLa cells. **A** BID KO HeLa cells were reconstituted with LKB1 WT, G135R, D176N, or D194N. Cell lysates were subjected to immunoblotting with the indicated antibodies. **B** Cell lysates from LKB1 (WT/G135R/D176N/D194N) reconstituted BID KO HeLa cells were immunoprecipitated with protein G-Sepharose beads using the indicated antibodies, and subjected to immunoblotting with the indicated antibodies. **C** LKB1 (WT, G135R, D176N, or D194N) reconstituted BID KO HeLa cells were treated with Fc-FasL (100 ng/mL) for 3 h, and then cell lysates were subjected to immunoblotting with the indicated antibodies. **D** Empty vector (EV) or LKB1 (WT or G135R) reconstructed BID KO HeLa cells were treated with Fc-FasL (100 ng/mL) for 0, 3 or 6 h, and then cell lysates were subjected to immunoblotting with the indicated antibodies. **E** Empty vector (EV) or LKB1 (WT, D176N, or D194N) reconstituted BID KO HeLa cells were treated with Fc-FasL (100 ng/mL) for 0, 3 or 6 h, and then cell lysates were subjected to immunoblotting with the indicated antibodies. **F** Empty vector (EV) or LKB1 (WT, G135R, D176N, or D194N) reconstituted BID KO HeLa cells were treated with Fc-FasL (0, 50, 100 ng/mL) for 12 h. Cell viability was determined by PMS/MTS assay. Data shown are the mean ± SD (n = 3). Statistical significance was tested using an unpaired Student’s t test; ***p < 0.001, (vs. EV cells), ###*p* < 0.001 (vs. LKB1 WT cells) (**G**) Empty vector (EV) or LKB1 (WT, G135R, D176N, or D194N) reconstituted BID KO HeLa cells were treated with Fc-FasL (0, 50, 100 ng/mL) for 12 h. Apoptotic cells were labeled with annexin V-FITC and PI for 15 min, and analyzed by FACS. Data is presented as FITC-PE fluorescence density plots. **H** Quantification of the percentage of V-positive cells shown in Fig. 5G. Data shown are the mean ± SD (n = 3). Statistical significance was tested using an unpaired Student’s t test; ***p < 0.001, (vs. EV cells), ###*p* < 0.001 (vs. LKB1 WT cells). **I** Quantification of the percentage of PI and annexin V double-positive cells shown in Fig. 5G. Data shown are the mean ± SD (n = 3). Statistical significance was tested using an unpaired Student’s t test; ***p < 0.001, (vs. EV cells), ###*p* < 0.001 (vs. LKB1 WT cells). **J** Quantification of the percentage of PI-positive/Annexin V-negative cells shown in Fig. 5G. Data shown are the mean ± SD (n = 3). Statistical significance was tested using an unpaired Student’s t test; ***p < 0.001, (vs. EV cells), ###*p* < 0.001 (vs. LKB1 WT cells). All data are representative of at least three biologically independent replicates.

In Figs. 1–4, we demonstrated the possibility that tLKB1 acts as a surrogate of Smac in the absence of cytosolic Smac, which maybe function as a backup mechanism to prevent failure of apoptosis induction. However, since the alternative function of LKB1 does not depend on its kinase activity, it is important to evaluate whether mutants lacking kinase activity found in PJS can still exert this function. We therefore established cell reconstitution of several LKB1 mutants (G135R, D176N, and D194N) found in PJS in BID KO HeLa cells (Fig. 5A). The binding affinity of these mutants to caspase-8 was not significantly different from that of WT (Fig. 5B). In addition, both D176N and D194N were cleaved by recombinant caspase-8 similarly to LKB1 WT, while G135R mutant could not be tested due to its instability and inability to be purified via affinity purification (Supplemental Fig. 6). However, interestingly, these LKB1 mutants failed to promote XIAP degradation and the processing of caspase-3 and caspase-8 (Fig. 5C–E), suggesting that structural alterations in these mutants may disrupt their ability to target XIAP. Consistent with these observations, reconstituted cells of these mutants remain strongly resistant to Fas-induced apoptosis, when analyzing both MTS assay and FACS analysis (Fig. 5F–J). Taken together, these observations suggest that the LKB1 mutants found in PJS failed to promote Fas-induced apoptosis following the XIAP degradation in BID KO HeLa cells.

## Discussion

**Fig. 6:**
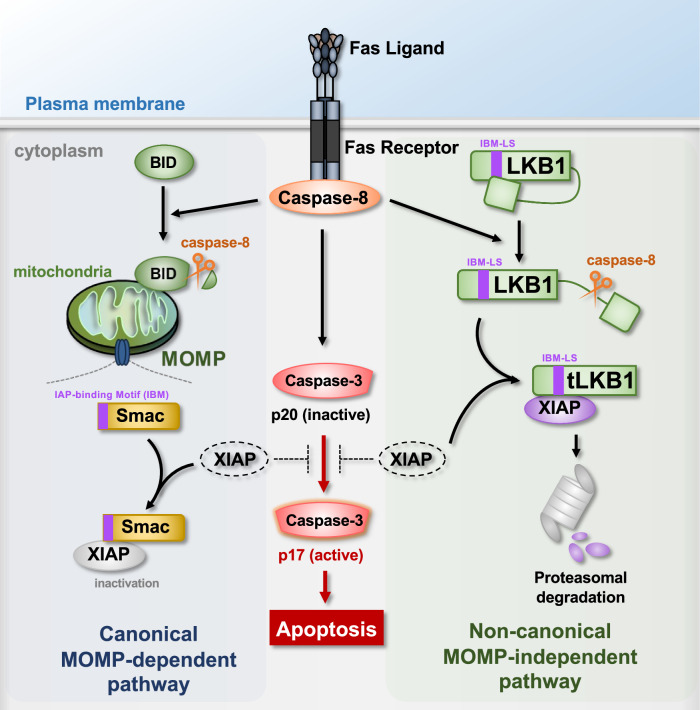
Schematic model to explain our study. Upon Fas activation, caspase-8 induces MOMP by cleaving BID, and then promotes the cytoplasmic release of Smac, resulting in XIAP inactivation and apoptosis (Left part). XIAP can also be degraded in a parallel pathway independent on MOMP and Smac release. In this case, caspase-8 cleaves LKB1 within its C-terminal region to release tLKB1. tLKB1 binds to and induces proteasomal degradation of XIAP. Lowering XIAP levels releases the break imposed on caspase-3 activation and apoptosis induction (Right part).

XIAP is a well-studied apoptosis inhibitor that directly inhibits caspase-3 activation, and it is widely known that overexpression of XIAP renders cancer cells resistant to apoptosis [50]. Several inhibitors of XIAP, such as Smac, ARTs, and Omi/Hrta1, have been identified [51–53]. Interestingly, these XIAP inhibitors are all mitochondrial proteins [51–53]. Therefore, MOMP-dependent release of the XIAP inhibitors into the cytoplasm is needed to inhibit XIAP. In this study, we have identified LKB1 as a novel negative regulator of XIAP that can work even in MOMP-deficient cancer cells. Our results demonstrated that LKB1 is truncated by caspase-8, and then tLKB1 interacts with XIAP, leading to the degradation of XIAP in MOMP-deficient cells (Fig. 6). When MOMP is normally induced, tBID promotes the release of Smac from the mitochondria into the cytoplasm, promoting the degradation of XIAP (Fig. 6) [54]. Therefore, LKB1 appears to play a role in Fas-induced apoptosis as a surrogate of Smac when MOMP is impaired (Fig. 6).

In regard to the degradation of XIAP, we found that the IBM-LS of LKB1 is required for the interaction with and degradation of XIAP. However, the detailed mechanism by which the C-terminal cleavage of LKB1 by caspase-8 enables XIAP to interact with the IBM-LS of LKB1 is unknown. In general, LKB1 interacts with STRAD and MO25 through its kinase domain, which is critical for the function of LKB1 as a kinase [55]. Meanwhile, it has been reported that the phosphorylation of LKB1 at Ser 428 is required for the LKB1-STRAD-MO25 binding, even though the phosphorylation site is located in the C-terminal domain [56]. These findings suggest that the C-terminal portion of LKB1 is structurally important in the formation of the LKB1-STRAD-MO25 complex. Therefore, C-terminal cleavage of LKB1 by caspase-8 would be expected to disrupt the LKB1-STRAD-MO25 complex, leading to loss of the kinase activity of LKB1. Taken together, the truncation of LKB1 by caspase-8 may act as a trigger to dissociate LKB1 from the STRAD-MO25 complex, and to allow the IBM-LS of LKB1 to bind to XIAP. Transient overexpression of the truncated LKB1 successfully induced the degradation of XIAP, but this was only partial compared to the degradation of XIAP induced by FasL treatment. Given that XIAP is degraded by activated caspase-3 as previous studies have shown [57, 58], FasL-induced activation of caspase-3 may assist the XIAP degradation mediated by tLKB1. In addition, overexpression of tLKB1 was sufficient to induce cell death.　However, the dead cells resulting from overexpression of tLKB1 contained a large number of PI-positive/Annexin V-negative cells, suggesting that necrotic cell death was induced. This observation is consistent with previous finding that overexpression of the mature form of HtrA2/Omi, the mitochondrial serine protease that induces degradation of XIAP, induces non-apoptotic cell death [59]. Furthermore, in several experiments where PI/Annexin staining was performed on LKB1-reconstituted cells, it was confirmed that reconstitution of LKB1 also increases the number of PI-positive/Annexin V-negative cells during FasL treatment.　These results suggest that the cell death induced by truncated LKB1 includes not only apoptosis but also necrotic cell death.

How tLKB1 can interact with XIAP and mediate its degradation is currently unclear. Involvement of the AVKI sequence of LKB1 as a potential IAP-binding motive is possible, but not necessarily the only way to interpret the results. The AVKI sequence of LKB1 contains a lysine residue instead of a proline residue present in Smac (AVKI instead of AVPI), but this is insufficient to disqualify the sequence as there is a precedent. The structure of the BIR2 domain of XIAP has been solved in complex with different peptides (AIAV, ATAA, SVPI, AVKI and AMRV), one of which displays a positively-charged residue at the same position [60]. However, the AVKI sequence of LKB1 is not exposed at the N-terminus, which is likely to interfere with the amino-terminal group heavily involved in interactions with both the BIR2 and BIR3 domains of XIAP [60, 61]. Moreover, the AVKI sequence of LKB1 is part of a structure of four anti-parallel beta-sheets [62], with lysine 78 contacting a phosphoryl group of ATP in the ATP-binding pocket (actually the same lysine of the kinase inactive mutant K78M). Taking account of these structural features, we certainly do not exclude another mechanism, yet to be elucidated, to explain the involvement of tLKB1 in XIAP inactivation.

While most of the mutations in LKB1 seen in PJS and the sporadic cancers are within the kinase domain of LKB1, there are also multiple mutations located in the C-terminal domain [63, 64]. Our findings, demonstrating that LKB1 truncated at the C-terminus induces apoptosis in a kinase activity-independent manner, may help to explain why C-terminal LKB1 mutations cause tumorigenesis. On the other hand, PJS LKB1 mutations abrogating kinase activity, such as G135R, D176N and D194N, were also unable to induce the degradation of XIAP and apoptosis. Considering that kinase activity of LKB1 is not required for degradation of XIAP, these observations suggest that these PJS mutations cause serious structural changes in LKB1, resulting in the loss of not only kinase activity but also the ability to degrade XIAP, both of which could be important features of tumor promotion. In any case, although further studies are needed to understand why the LKB1 mutants that has the mutation in its kinase domain fail to degrade XIAP, our results could lead to a better understanding of the antitumor functions of LKB1.

## Materials and methods

### Reagents and plasmids

Human Fc-Fas ligand was produced and purified as described previously [44, 65].　The information of other reagents and antibodies is written in Supplemental Table 1. cDNAs encoding human LKB1 (WT and other mutations) were obtained by performing PCR, and inserted into pMXs plasmid. cDNAs encoding human LKB1, XIAP and Caspase-8 (p43 C360A) was obtained by performing PCR, and inserted into pcDNA with FLAG or 6 x myc or HA tag plasmid. Plasmid transfection was performed using Polyethylenimine “Max” (PEI-MAX, Polysciences, Pennsylvania, United States), according to the manufacturer’s instructions.

### Cell lines

HeLa cells and HEK293A purchased from RIKEN BRC CELL BANK (RCB No. 0007) and Invitrogen, respectively, and HT1080 cells obtained from ATCC were grown in Dulbecco’s Modified Eagle Medium (DMEM), 10% heat-inactivated fetal bovine serum (FBS), and 1% penicillin-streptomycin solution, at 37 °C under a 5% CO_2_ atmosphere.

### Generation of knockout cell lines

*BID or Caspase-3* knockout cells were generated using the CRISPR/Cas9 system. Guide RNAs (gRNAs) were designed to target a region in the exon 2 of *BID* gene (5’-CGCAGAGAGCTGGACGCACTGGG-3’) and a region in the exon 5 of Caspase-3 gene (5’-CATACATGGAAGCGAATCAATGG-3’) using CRISPRdirect [66]. gRNA-encoding oligonucleotide was cloned into lentiCRISPRv2 plasmid (addgene) [67], and knockout cells were established as previously described [68]. To determine the mutations of *BID or Caspase-3* in cloned cells, genomic sequence around the target region was analyzed by PCR-direct sequencing using extracted DNA from each clone as a template and the following primers: For determination of BID mutation; 5’-GCTTCCTGACTCCCCTTTCC-3’ and 5’-ATTCTTCCCAAGCGGGAGTG-3’. For determination of Caspase-3 mutation; 5’- CATACATGGAAGCGAATCAA-3’ and 5’-TTGATTCGCTTCCATGTATG-3’.

### Generation of reconstituted cells

Reconstituted HeLa cells were generated by retroviral transduction as previously described [69]. A packaging cell line Phoenix-AMPHO was transfected with pMXs-IH inserted with LKB1 WT, LKB1 K78M mutant, LKB1 4DE and similar mutants, LKB1 ΔIBM-LS mutant and PJS mutants. After 48 h, the growth medium containing retrovirus was collected. HeLa cells were incubated with the virus-containing medium with 10 μg/mL polybrene for 48 h, and uninfected cells were eliminated by hygromycin selection.

### Colorimetric cell viability assay

Cells were seeded on 96-well plates. After indicated stimulation or treatment, cell viability was determined using Cell Titer 96 Cell Proliferation Assay (Promega, Wisconsin, United States), according to the manufacturer’s protocol [70]. The absorbance was read at 492 nm using a microplate reader. Data are normalized to control (100%) without stimulus, unless noted otherwise.

### FACS analysis

FACS analysis was performed as described previously [71]. For annexin V and propidium iodide (PI) staining, HeLa cells were labeled with annexin V-FITC (MBL, Tokyo, Japan) and PI for 15 min. Fluorescent cells were detected by CytoFLEX (Beckman Coulter, California, United States), and apoptotic cells were analyzed by using CytoExpert (Beckman Coulter).

### Mitochondrial membrane potential assay

Mitochondrial membrane potential was measured by JC-1 MitoMP Detection Kit (MT09) (Wako, Osaka, Japan) according to the manufacturer’s instructions [70]. Cells were treated with indicated reagents and then incubated with 2 µM JC-1 dye for 30 min at 37 °C. After incubation, the cells were measured for fluorescence intensity by SPECTRA max GEMINI XPS (Molecular Devices) as described previously. JC-1 dye is captured by mitochondria and exhibits strong red fluorescence. However, loss of mitochondrial membrane potential (MMP) blocks the capture, and then JC-1 exhibits green fluorescence. Thus, reduction of MMP can be measured by the ratio of the red/green fluorescence.

### Immunoblot

Cells were lysed with DISC lysis buffer (1% Triton X-100, 150 mM NaCl, 20 mM Tris-HCl (pH 7.4), 1% protease inhibitor cocktails (Nacalai Tesque, Kyoto, Japan), and 10% glycerol). After centrifugation at 15,000 rpm for 15 min, the cell extracts were resolved by SDS-PAGE, and subjected to immunoblot analysis as previously described [72].

### Immunoprecipitation

Immunoprecipitation was carried out with a modified procedure as previously described [73]. After the indicated treatment, cells were lysed in ice-cold DISC Lysis IGEPAL-1 buffer [25 mM Hepes (pH 7.5), 150 mM NaCl, 1% NP-40 (IGEPAL CA-630), 10% glycerol, 1 mM DTT, and 1% protease inhibitor cocktails] for 15 min. Cell lysates were centrifuged at 4 °C at 15,000 rpm for 15 min. The cell extracts were immunoprecipitated with 20 μl anti-FLAG–tagged agarose beads or protein G-Sepharose beads (Amersham Biosciences, Uppsala, Sweden) for 12 h at 4 °C with the indicated antibodies. The beads were washed four times with the same buffer before analysis by SDS-PAGE or affinity purification.

### Affinity purification of FLAG-LKB1

Immunoprecipitated FLAG-LKB1 binding to FLAG-tagged agarose beads was eluted with 3xFLAG peptide (Sigma-Aldrich, Missouri, United states) for 1 h at 4 °C. After centrifugation, the supernatant was obtained as the purified protein.

### In vitro caspase assay

Affinity-purified LKB1 and Active recombinant Human Caspase-8, Caspase-3, Caspase-9 (Enzo Life Sciences, New York, United States) were reacted in Caspase buffer (50 mM HEPES-KOH (pH 7.5), 100 mM NaCl, 0.5% CHAPS, 10% Glycerol, 10 mM DTT) at 37 °C for 1 h.

### Colorimetric caspase assay

Recombinant active-caspase-8, active-caspase-3, or active-caspase-9 was mixed with caspase reaction buffer (10 mM Tris-HCl pH 7.4, 150 mM NaCl, 0.1% CHAPS, 2 mM MgCl2, 5 mM EGTA, and 1 mM DTT) with the caspase-8-specific substrate IEDT-pNA, caspase-3-specific substrate DEVD-pNA, or caspase-9-specific substrate LEHD-pNA, respectively, at a final concentration of 100 µM and incubated at 37 °C for 1 h. Its activity was measured using a microplate reader and the absorbance was read at 405 nm.

### Statistical analysis

The value was expressed as the mean ± standard error of the mean (S.E.M.) using Prism software (GraphPad). All experiments were repeated at least three independent times. Two groups were compared using student’s t test. Data were considered significant when *p < 0.05, **p < 0.01, ***p < 0.001.

**Table:** 

Information of antibodies	
Rabbit polyclonal anti-BID (Human Specific)	Cell Signaling Technology
Rabbit monoclonal anti-PARP (clone 46D11)	Cell Signaling Technology
Rabbit monoclonal anti-STK11 (clone 27D10)	Cell Signaling Technology
Rabbit monoclonal anti-XIAP (clone D2Z8W)	Cell Signaling Technology
Rabbit monoclonal anti- Caspase-8 (clone D35G2)	Cell Signaling Technology
Rabbit monoclonal anti-HA (clone-C29F4)	Cell Signaling Technology
Rabbit monoclonal anti-phospho-AMPK (clone-T172)	Cell Signaling Technology
Mouse monoclonal anti-AMPK α (clone-F6)	Cell Signaling Technology
Mouse monoclonal anti-Caspase-8 (clone 12F5)	Enzo Life Sciences
Mouse monoclonal anti-αTubulin (clone B-7)	Santa cruz biotechnology
Mouse monoclonal anti-β-Actin (clone C4)	Santa cruz biotechnology
Mouse monoclonal anti-Caspase-3 p17 (clone B-4)	Santa cruz biotechnology
Mouse monoclonal anti-STK11 (clone E-9)	Santa cruz biotechnology
Mouse monoclonal anti-STK11 (clone Ley 37D/G6)	Santa cruz biotechnology
Mouse monoclonal anti-XIAP (E-2)	Santa cruz biotechnology
Mouse polyclonal anti-Myc (Human Specific)	Santa cruz biotechnology
Rabbit monoclonal anti-FLAG	Sigma Aldrich
Information of reagents	
protein G-Sepharose beads	Amersham Biosciences
Active recombinant-caspase-8	BioVision
caspase-8-specific substrate IEDT-pNA	BioVision
caspase-3-specific substrate DEVD-pNA	BioVision
caspase-9-specific substrate LEHD-pNA	BioVision
MG132	Enzo Life Science
Active recombinant-caspase-3	Enzo Life Science
Active recombinant-caspase-9	Enzo Life Science
Annexin V-FITC	MBL
Propidium iodide	Nacalai tesque
Protease inhibitor cacktails	Nacalai tesque
z-VAD-fmk	peptide institute
PEI-MAX	Polyscience
Cell Titer 96 Cell Proliferation Assay kit	Promega
Bafilomycin A1	Santa cruz biotechnology
3xFLAG peptide	Sigma-Aldrich
Anti-FLAG-Tagged beads	Sigma-Aldrich
JC-1 MitoMP Detection Kit (MT09)	Wako

## Supplementary information

STK11-XIAP paper supplemental figure

Full-length amd uncropped WB data

## Data Availability

The data presented in this study are available within the article.
