# Supplementary material for: Truncated LKB1 nonenzymatically enhances Fas-induced apoptosis by acting as a surrogate of Smac
Source: Cell Death Discov. 2025 Jun 21;11:285. doi: 10.1038/s41420-025-02570-1 (PMC12182575; doi:10.1038/s41420-025-02570-1)
Supplement: Supplementary file 1 — STK11-XIAP paper supplemental figure [file 41420_2025_2570_MOESM1_ESM.pdf]

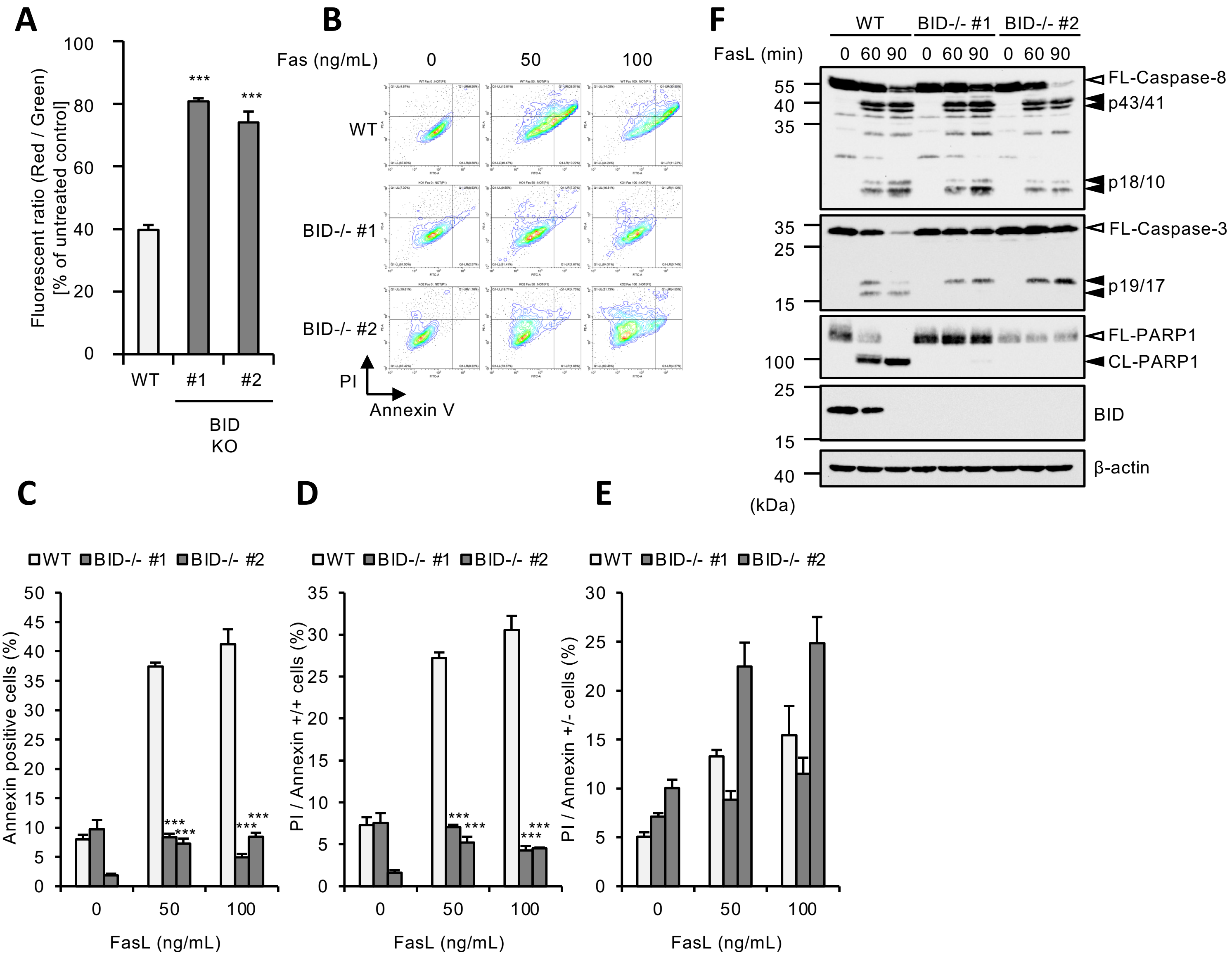

Figure S1. BID is required for Fas-induced apoptosis in HeLa cells

(A) WT and BID KO HeLa cells are treated with Fc-FasL (100 ng/mL) for 12 hours. Mitochondrial membrane potential was determined by JC-1 assay. Data shown are the mean ± SD (n = 3). Statistical significance was tested using an unpaired Student's t-test; *** $p < 0.001$, (vs. control cells). (B) WT and BID KO HeLa cells are treated with Fc-FasL (0, 50, 100 ng/mL) for 12 hours. Apoptotic cells were labeled with annexin V-FITC and PI for 15 min and analyzed by FACS. Data is presented as FITC-PE fluorescence density plots. (C) Quantification of the percentage of Annexin V-positive cells shown in Fig. S1B. Data shown are the mean ± SD (n = 3). Statistical significance was tested using an unpaired Student's t-test; *** $p < 0.001$, (vs. WT cells). (D) Quantification of the percentage of PI-Annexin double positive cells shown in Fig. S1B. Data shown are the mean ± SD (n = 3). Statistical significance was tested using an unpaired Student's t-test; *** $p < 0.001$, (vs. WT cells). (E) Quantification of the percentage of PI-positive/Annexin V-negative cells shown in Fig. S1B. Data shown are the mean ± SD (n = 3). (F) WT and BID KO HeLa cells are treated with Fc-FasL (100 ng/mL) for indicated periods, and then cell lysates were subjected to immunoblotting with the indicated antibodies. All data are representative of at least three biologically independent replicates.

Supplemental Fig. 1

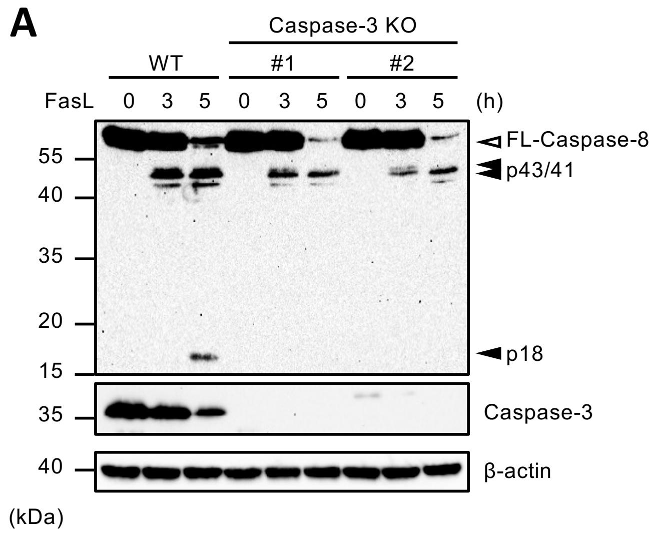

Figure S2 : Caspase-3 promotes the processing of caspase-8 to p18

(A) WT and Caspase-3 KO HT1080 cells are treated with Fc-FasL (100 ng/mL) for indicated periods, and then cell lysates were subjected to immunoblotting with the indicated antibodies.

Supplemental Fig. 2

A B

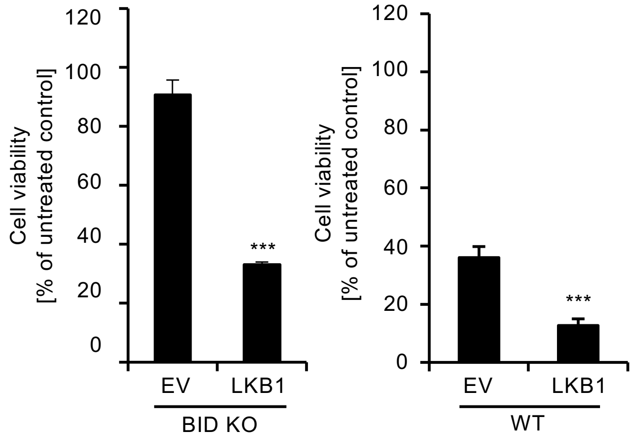

Figure S3. LKB1 promotes Fas-induced apoptosis eve in BID-expressing cells

(A) Empty vector (EV) or LKB1 WT reconstituted BID KO HeLa cells were treated with Fc-FasL (100 ng/mL) for 13 h. Cell viability was determined by PMS/MTS assay. Data shown are the mean ± SD (n = 3). Statistical significance was tested using an unpaired Student’s t-test; *** $p < 0.001$, (vs. EV cells) (B) Empty vector (EV) or LKB1 WT reconstituted HeLa cells were treated with Fc-FasL (100 ng/mL) for 13 h. Cell viability was determined by PMS/MTS assay. Data shown are the mean ± SD (n = 3). Statistical significance was tested using an unpaired Student’s t-test; *** $p < 0.001$, (vs. EV cells) . All data are representative of at least three biologically independent replicates.

Supplemental Fig. 3

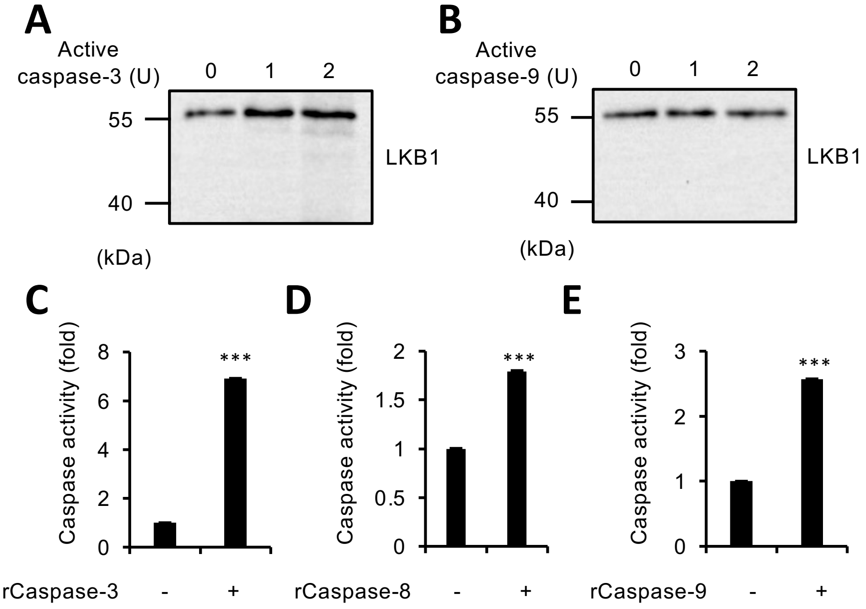

Figure S4. LKB1 is not cleaved by caspase-3 or caspase-9

(A) FLAG-LKB1, affinity-purified from 293A cells overexpressing FLAG-LKB1, was reacted with recombinant active caspase-3 (0, 1, 2 units), and subjected to immunoblotting with the indicated antibodies. (B) FLAG-LKB1, affinity-purified from 293A cells overexpressing FLAG-LKB1, was reacted with recombinant active caspase-9 (0, 1, 2 units), and subjected to immunoblotting with the indicated antibodies. (C) Recombinant Caspase-3 activity was measured by the Colorimetric caspase-3 assay. Data are shown as the ratio of caspase-3 activity versus the corresponding controls. Data shown are the mean ± S.D. Statistical significance was tested using an unpaired Student's t-test; *** $p < 0.001$, (vs. control). (D) Recombinant Caspase-8 activity was measured by the Colorimetric caspase-3 assay. Data are shown as the ratio of caspase-8 activity versus the corresponding controls. Data shown are the mean ± S.D. Statistical significance was tested using an unpaired Student's t-test; *** $p < 0.001$, (vs. control). (E) Recombinant Caspase-9 activity was measured by the Colorimetric caspase-3 assay. Data are shown as the ratio of caspase-9 activity versus the corresponding controls. Data shown are the mean ± S.D. Statistical significance was tested using an unpaired Student's t-test; *** $p < 0.001$, (vs. control). All data are representative of at least three biologically independent replicates.

Supplemental Fig. 4

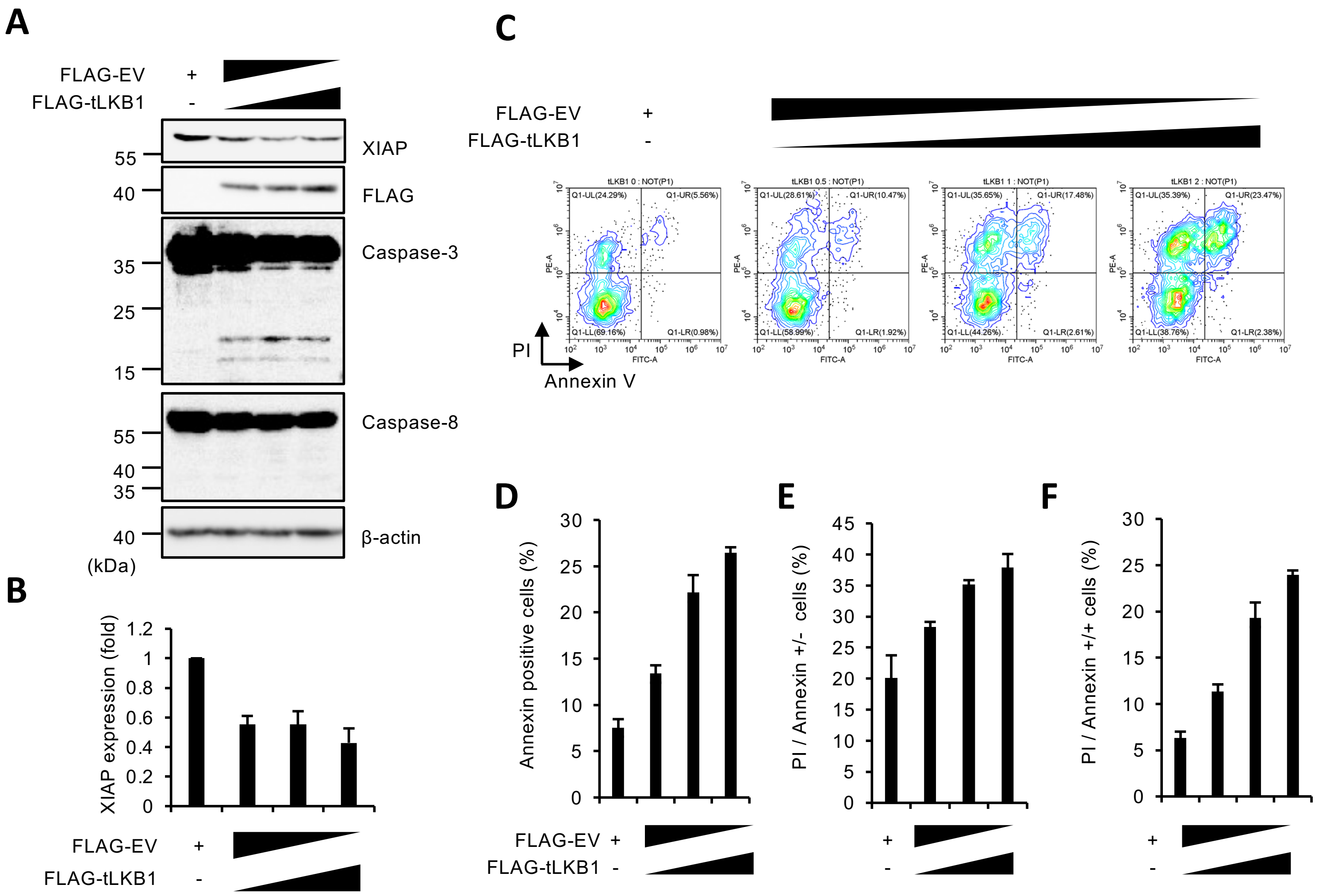

Figure S5. Transient expression of truncated LKB1 promotes the degradation of XIAP and cell death

(A) BID KO HeLa cells were transfected with increasing amounts of FLAG-LKB1 (Δ382) plasmid (0, 0.5, 1, or 2 µg / well), complemented with empty vector to a total plasmid DNA amount of 2 µg / well. After 24 hours, the cell lysates were subjected to immunoblotting with the indicated antibodies. (B) Quantification of the expression of XIAP in Fig. S3A. Data shown are the mean ± SD (n = 3). (C) BID KO HeLa cells were transfected with increasing amounts of FLAG-LKB1 (Δ382) plasmid (0, 0.5, 1, or 2 µg / well), complemented with empty vector to a total plasmid DNA amount of 2 µg / well. After 24 hours, apoptotic cells were labeled with annexin V-FITC and PI for 15 min and analyzed by FACS. Data is presented as FITC-PE fluorescence density plots. (D) Quantification of the percentage of Annexin V-positive cells shown in Fig. S3C. Data shown are the mean ± SD (n = 3). (E) Quantification of the percentage of PI-Annexin double positive cells shown in Fig. S3C. Data shown are the mean ± SD (n = 3). (F) Quantification of the percentage of PI-positive/Annexin V-negative cells shown in Fig. S3C. Data shown are the mean ± SD (n = 3). All data are representative of at least three biologically independent replicates.

Supplemental Fig. 5

A

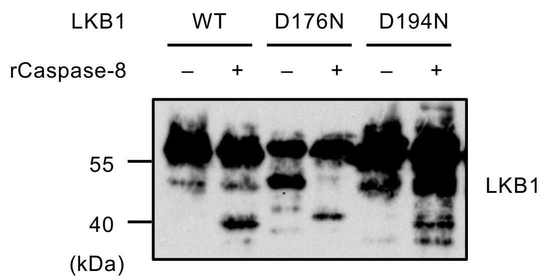

Figure S6. D176N and D194N mutants are also cleaved by caspase-8

(A) FLAG-LKB1 (WT, D176N, D194N), affinity-purified from 293A cells overexpressing FLAG-LKB1, were reacted with recombinant active caspase-8 (0.5 units), and subjected to immunoblotting with the indicated antibodies. This data is representative of at three biologically independent replicates.

Supplemental Fig. 6
