# Supplementary material for: Truncated LKB1 nonenzymatically enhances Fas-induced apoptosis by acting as a surrogate of Smac
Source: Cell Death Discov. 2025 Jun 21;11:285. doi: 10.1038/s41420-025-02570-1 (PMC12182575; doi:10.1038/s41420-025-02570-1)
Supplement: Supplementary file 2 — Full-length amd uncropped WB data [file 41420_2025_2570_MOESM2_ESM.pdf]

Full-length and uncropped western blot for Figure 1A

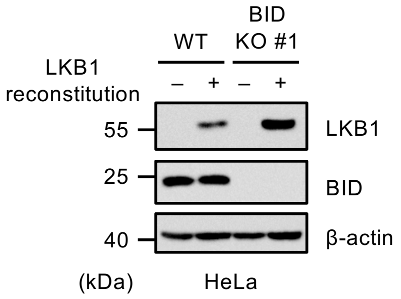

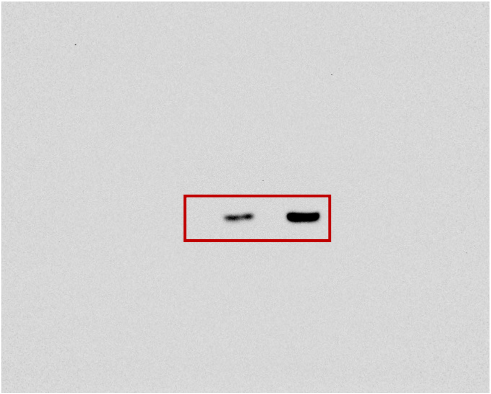

Figure 1A LKB1

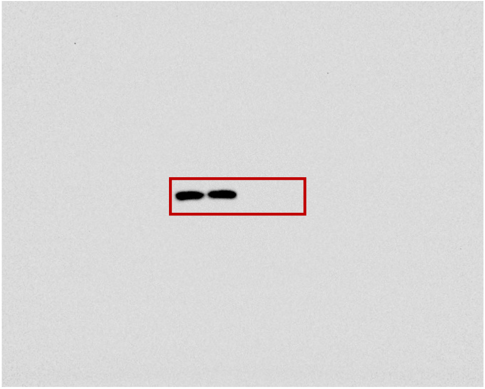

Figure 1A BID

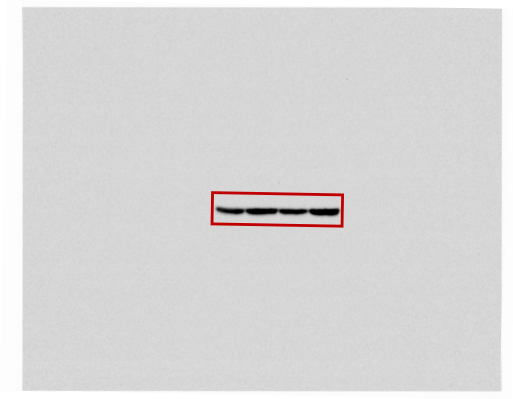

Figure 1A β-actin

Full-length and uncropped western blot for Figure 1B

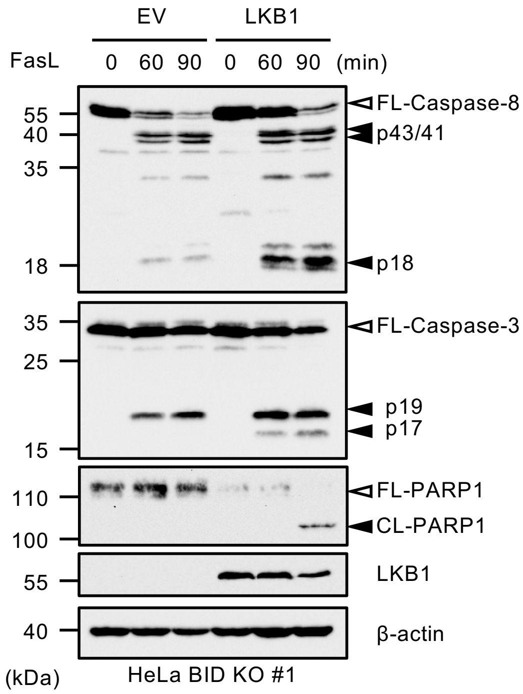

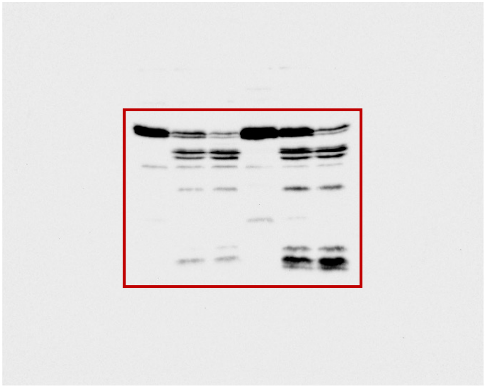

Figure 1B Caspase-8

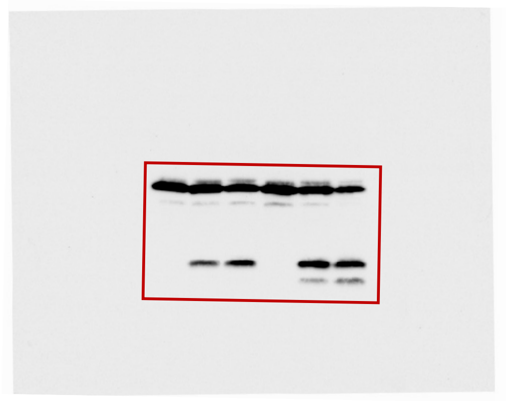

Figure 1B Caspase-3

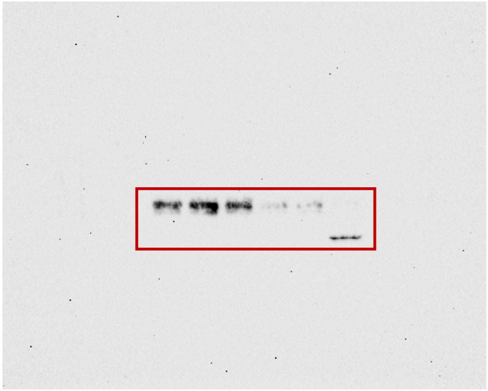

Figure 1B Cleaved PARP (IB : PARP-1)

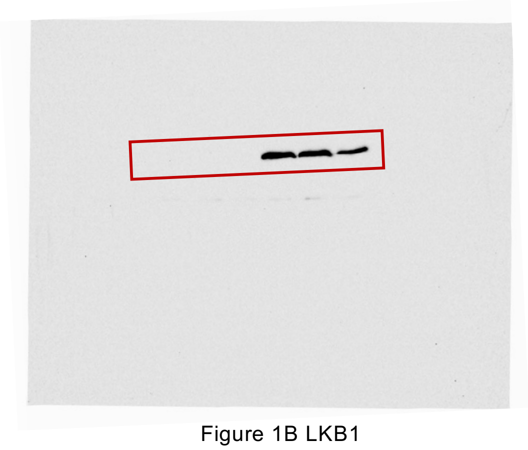

Figure 1B LKB1

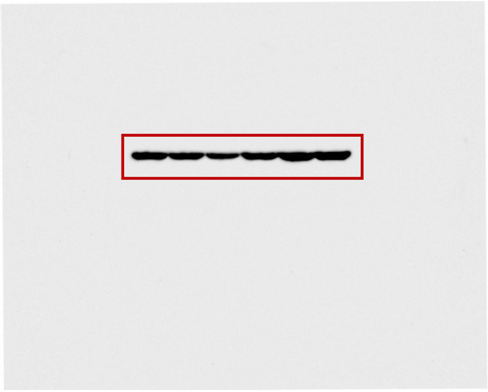

Figure 1B β-actin

Full-length and uncropped western blot for Figure 1H

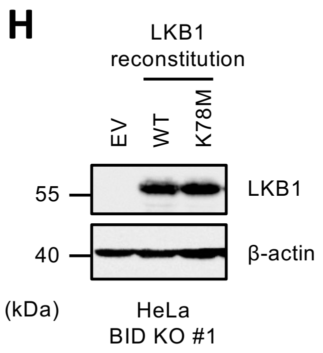

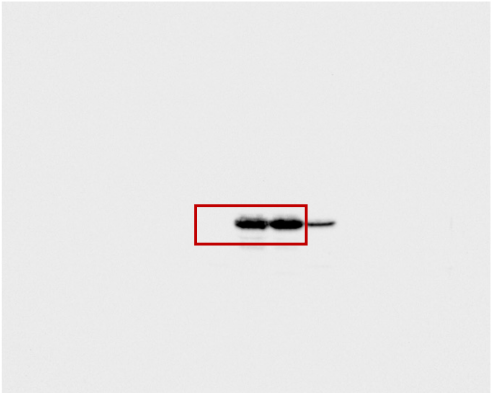

Figure 1H LKB1

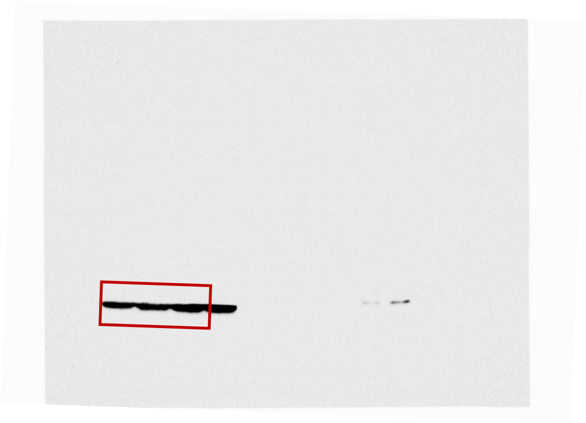

Figure 1H β-actin

Full-length and uncropped western blot for Figure 1I

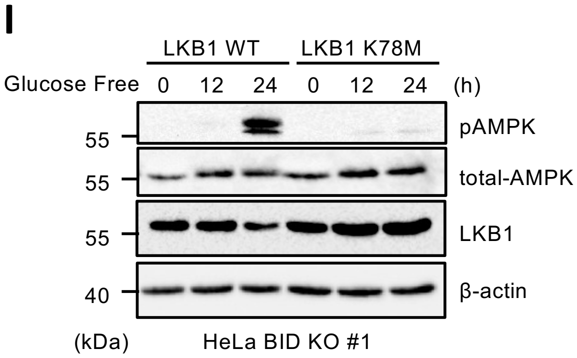

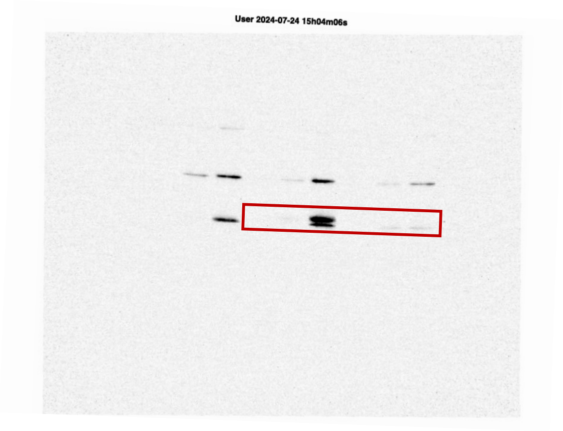

Figure 1I pAMPK

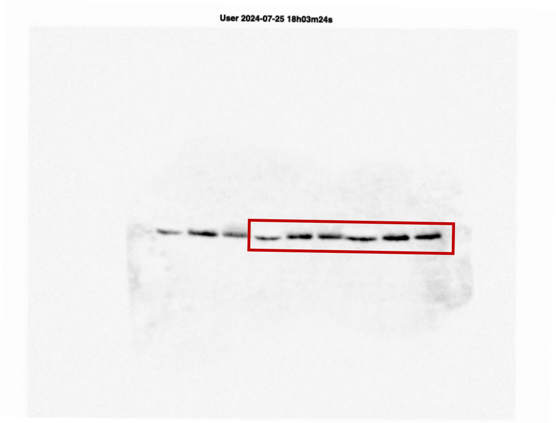

Figure 1I total-AMPK

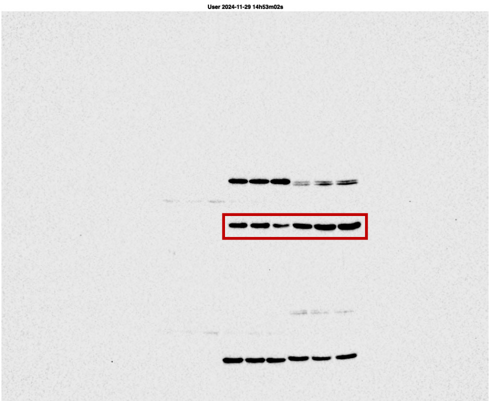

Figure 1I LKB1

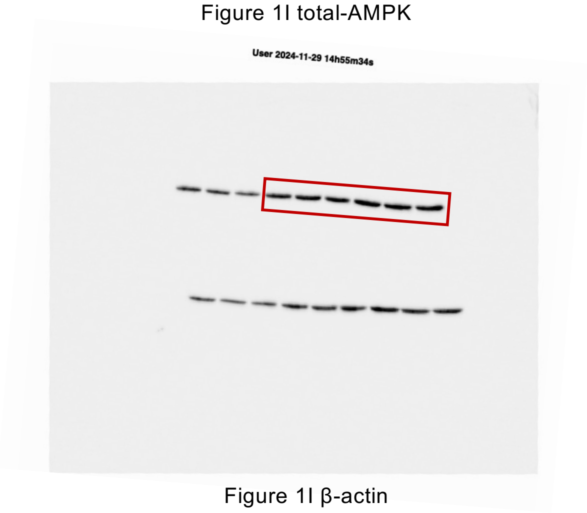

Figure 1I β-actin

Full-length and uncropped western blot for Figure 2A

A

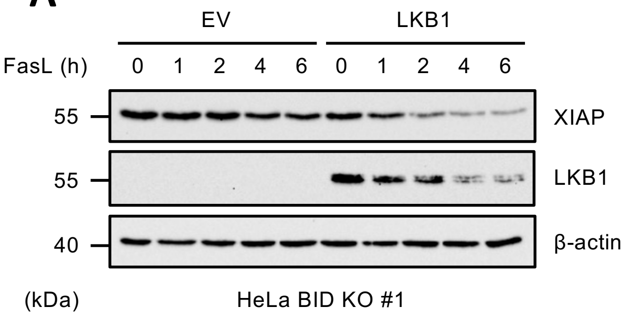

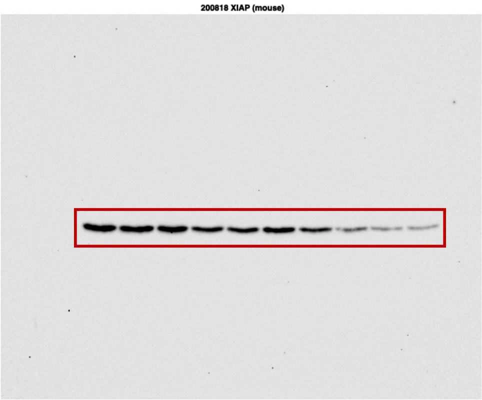

Figure 2A XIAP

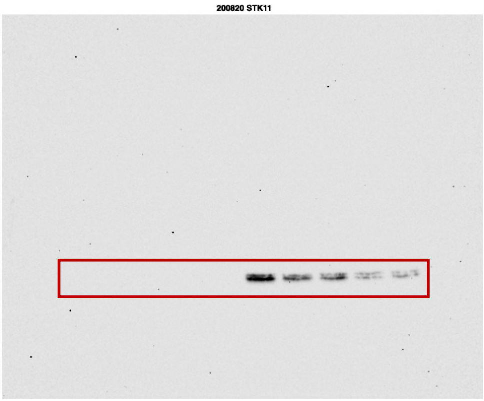

Figure 2A LKB1

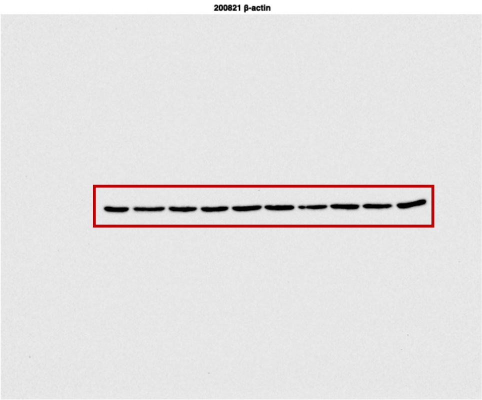

Figure 2A β-actin

Full-length and uncropped western blot for Figure 2B

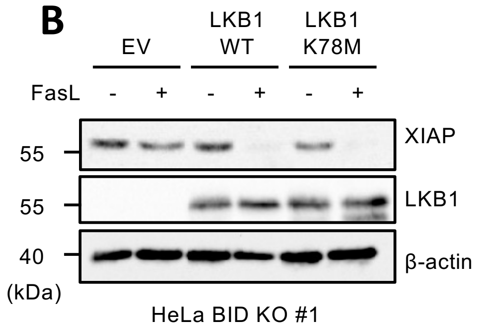

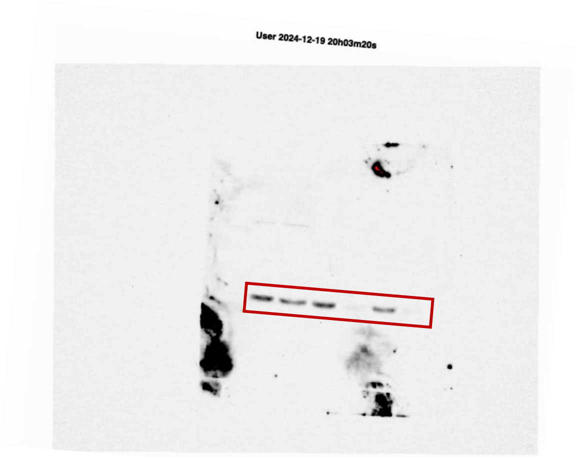

Figure 2B XIAP

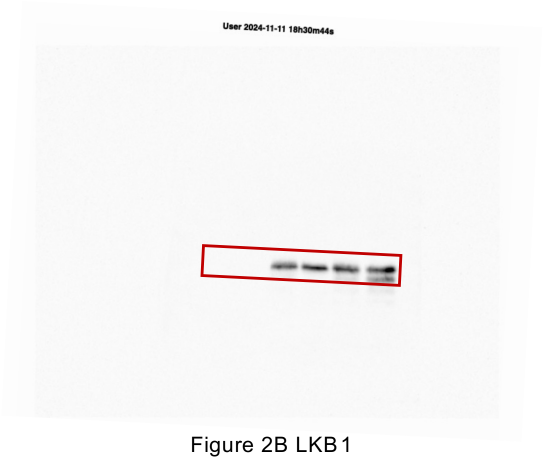

Figure 2B LKB1

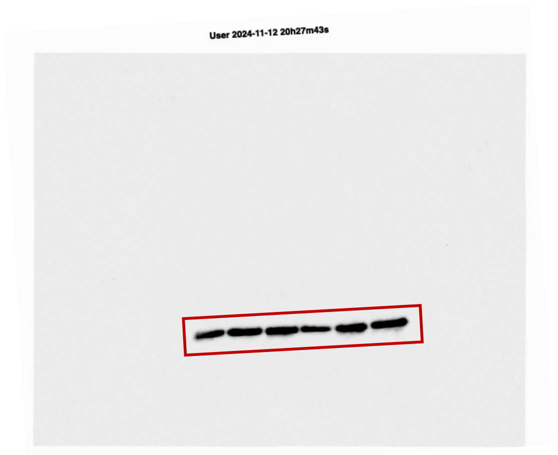

Figure 2B β-actin

Full-length and uncropped western blot for Figure 2C

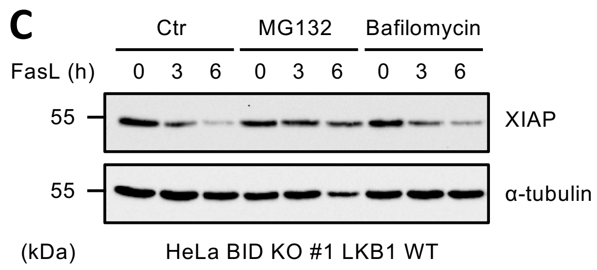

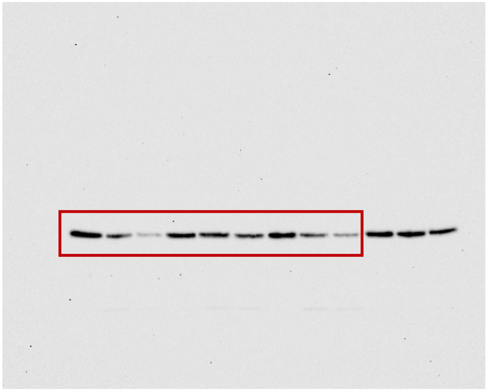

Figure 2C XIAP

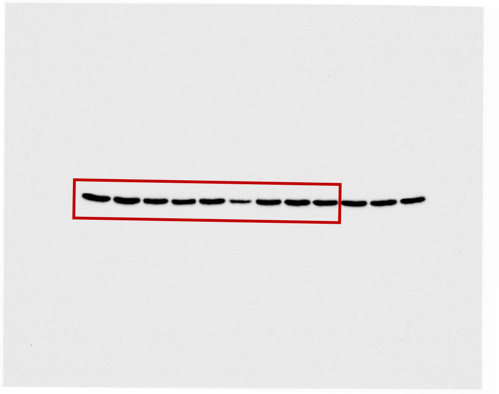

Figure 2C β-actin

Full-length and uncropped western blot for Figure 2D

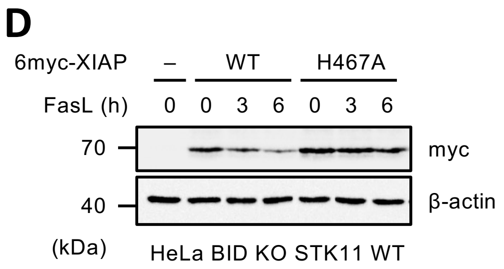

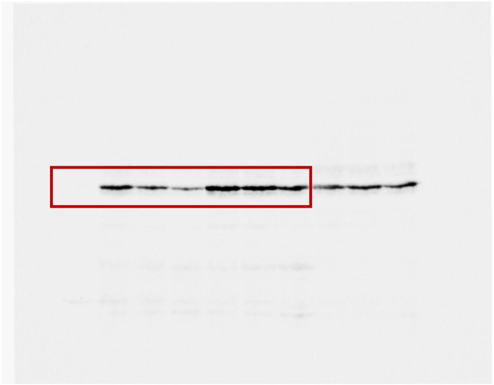

Figure 2D myc

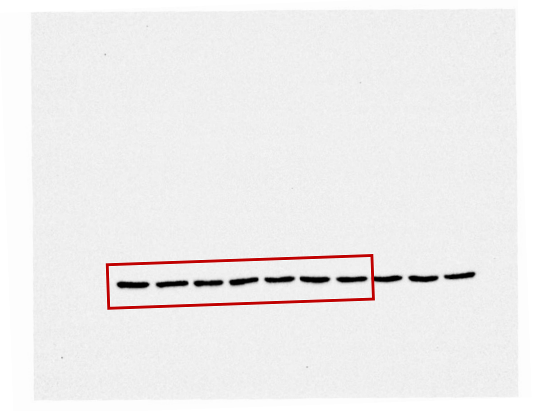

Figure 2D β-actin

Full-length and uncropped western blot for Figure 2E

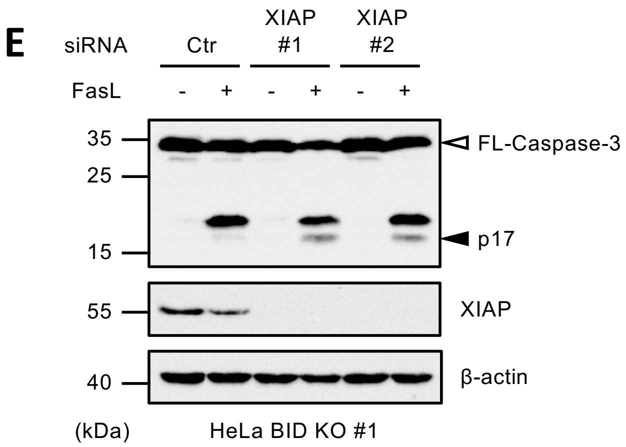

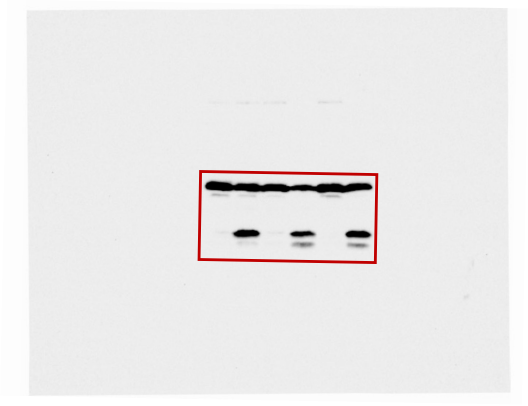

Figure 2E Caspase-3

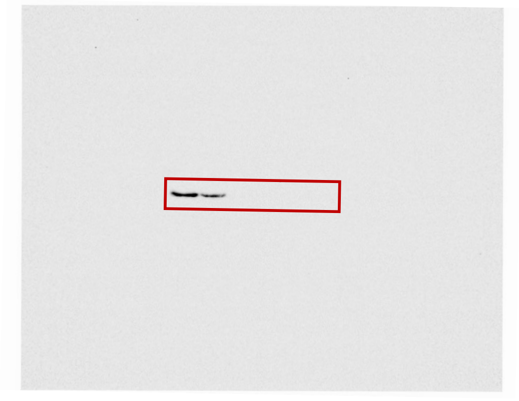

Figure 2E XIAP

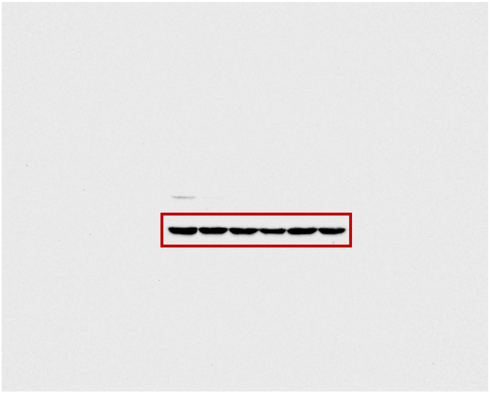

Figure 2E β-actin

Full-length and uncropped western blot for Figure 3A

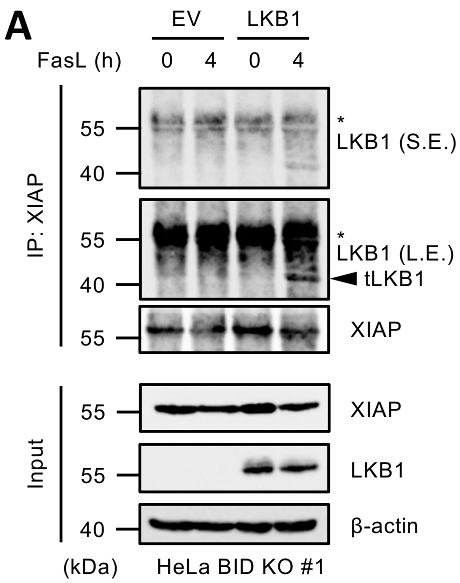

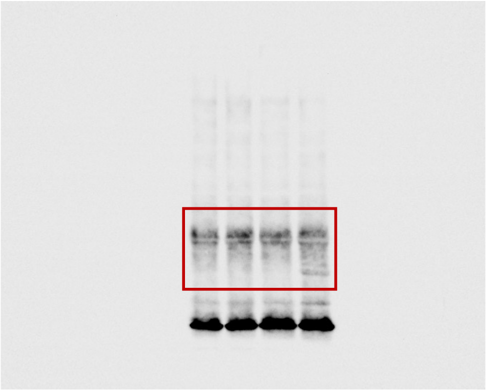
Figure 3A LKB1 (S.E.) (IP)

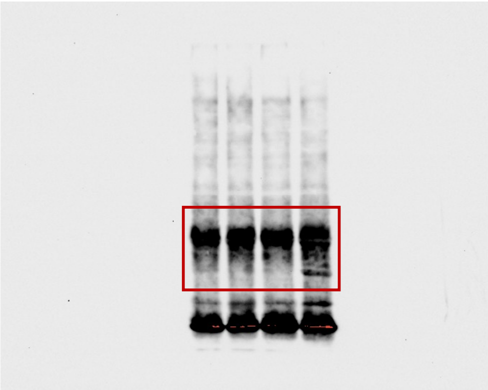
Figure 3A LKB1 (L.E.) (IP)

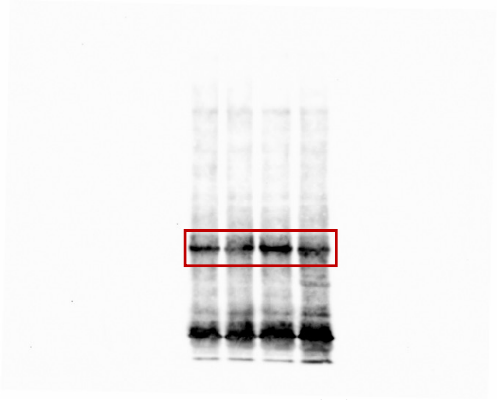
Figure 3A XIAP (IP)

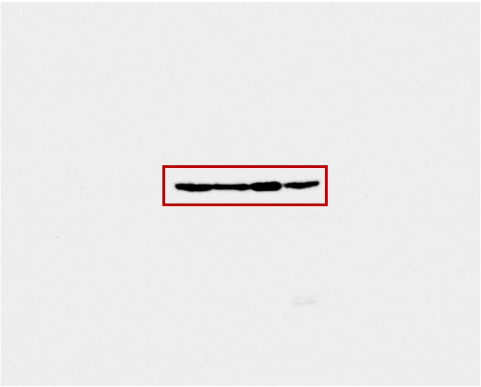
Figure 3A XIAP (input)

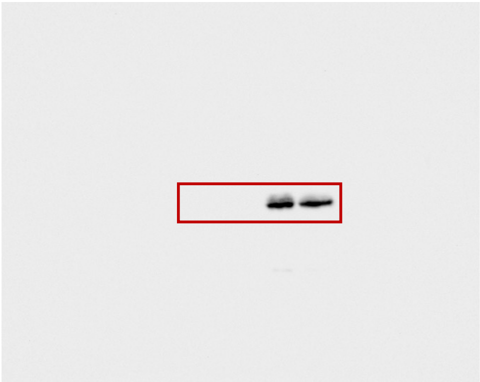
Figure 3A LKB1 (input)

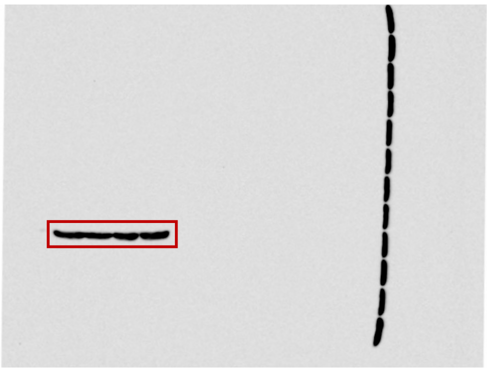
Figure 3A β-actin (input)

Full-length and uncropped western blot for Figure 3B

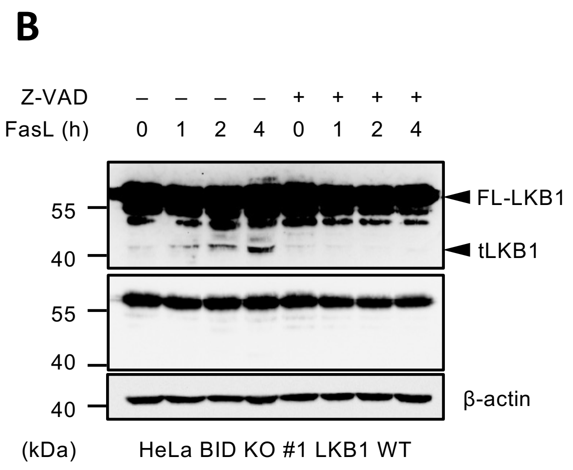

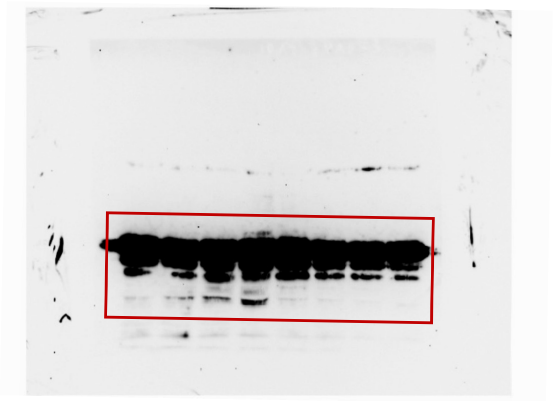

Figure 3B LKB1 (L.E.)

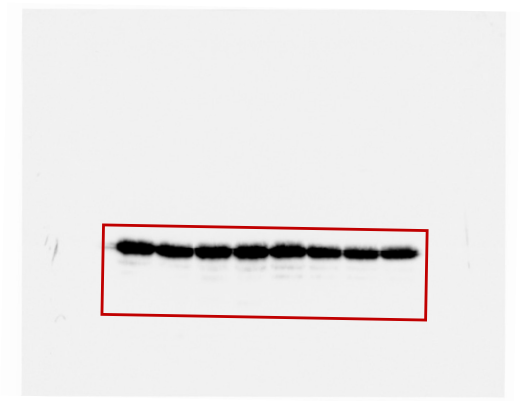

Figure 3B LKB1 (S.E.)

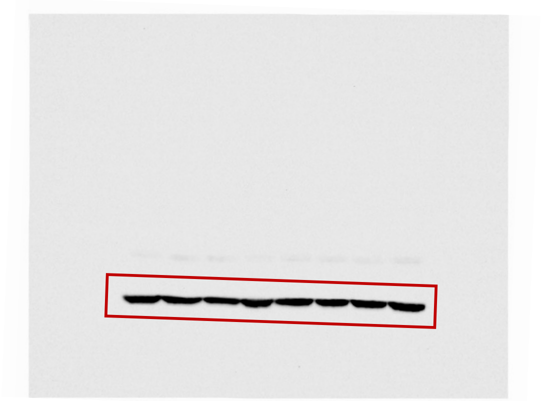

Figure 3B input

Full-length and uncropped western blot for Figure 3C

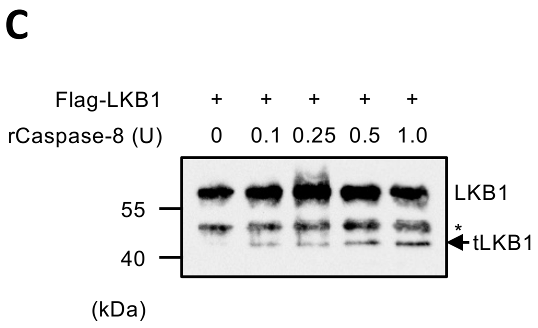

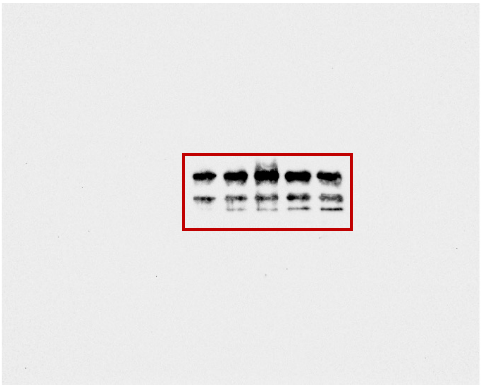

Figure 3C LKB1

Full-length and uncropped western blot for Figure 3D

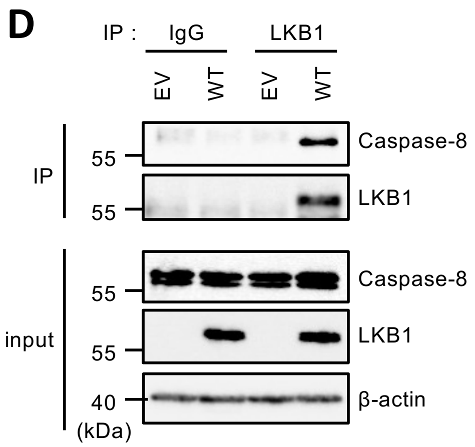

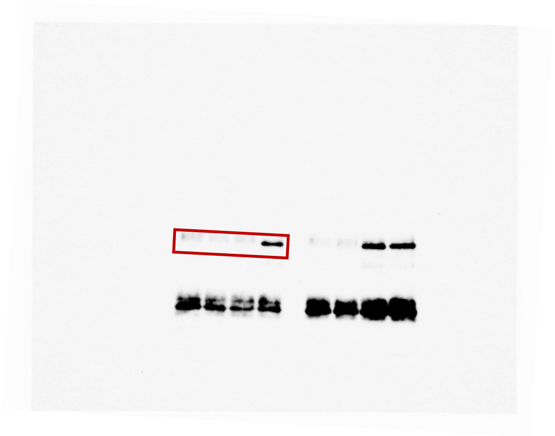

Figure 3D Caspase-8 (IP)

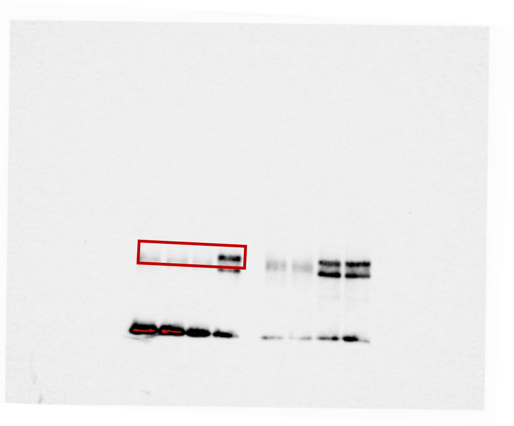

Figure 3D LKB1 (IP)

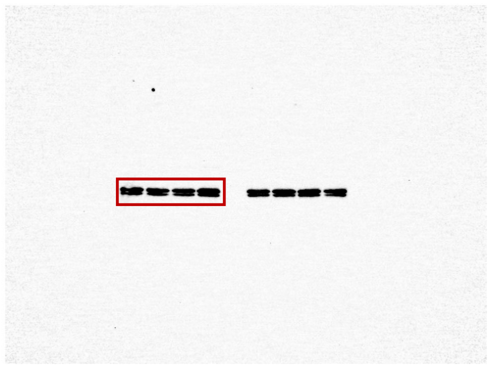

Figure 3D Caspase-8 (input)

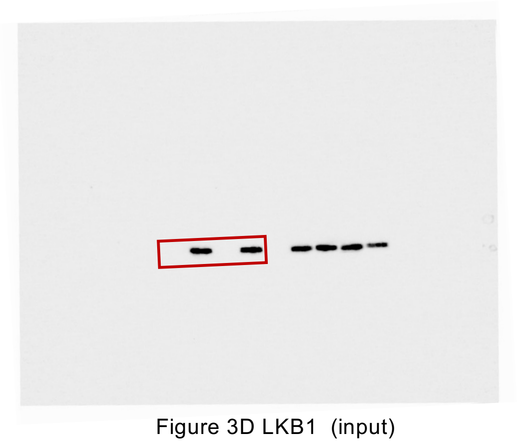

Figure 3D LKB1 (input)

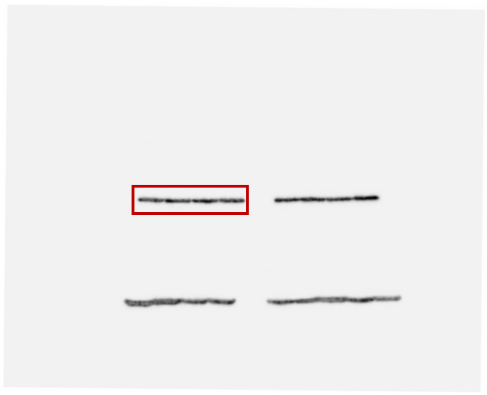

Figure 3D β-actin (input)

Full-length and uncropped western blot for Figure 3E

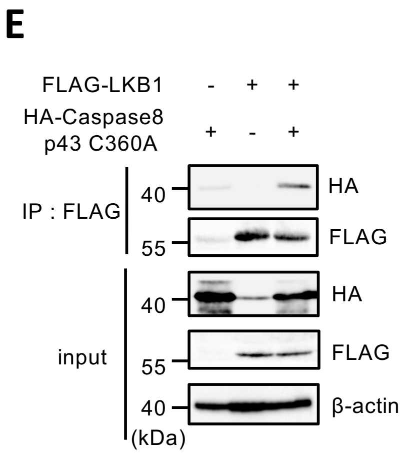

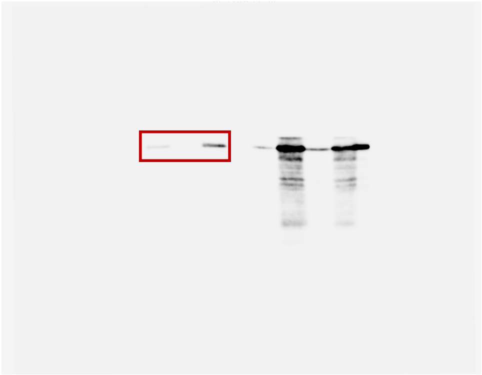

Figure 3E HA (IP)

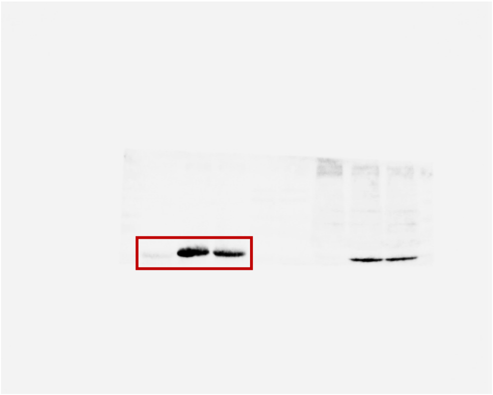

Figure 3E FLAG (IP)

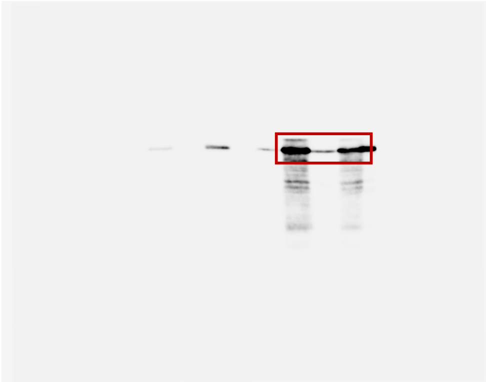

Figure 3E HA (input)

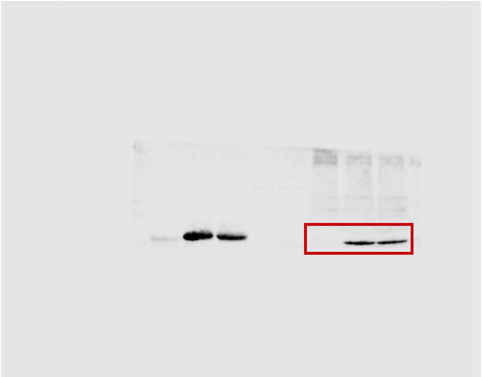

Figure 3E FLAG (input)

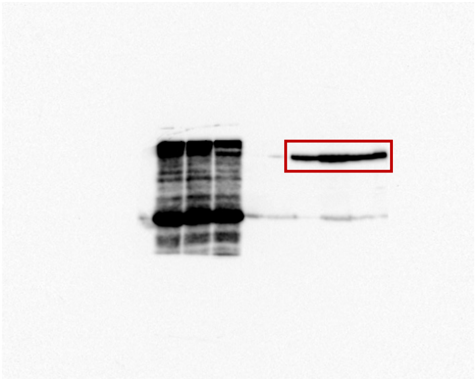

Figure 3E β-actin (input)

Full-length and uncropped western blot for Figure 3F

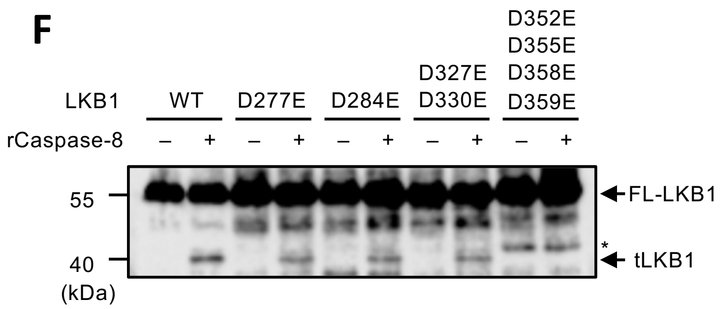

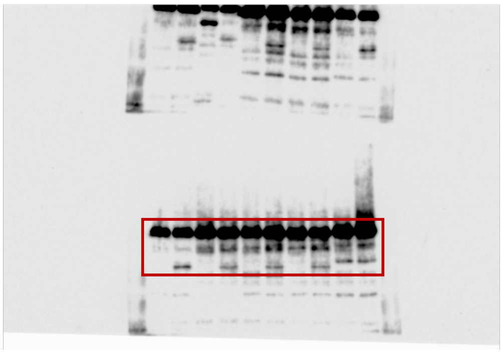

Figure 3F LKB1

Full-length and uncropped western blot for Figure 3G

G

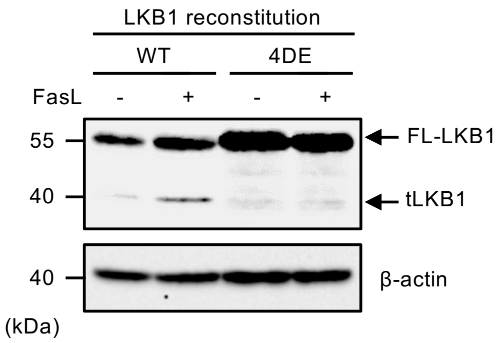

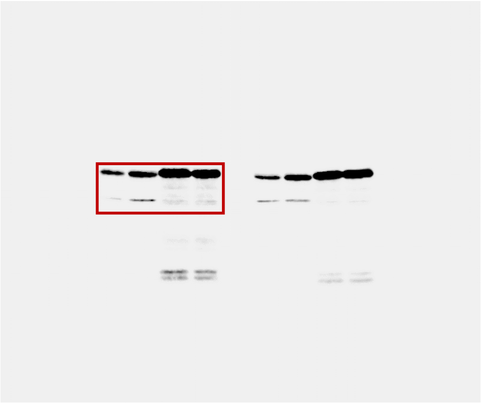

Figure 3G LKB1

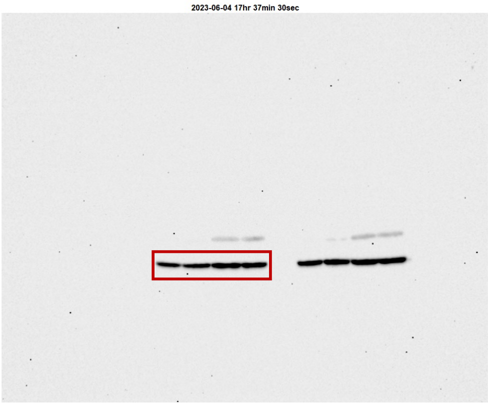

Figure 3G β-actin

Full-length and uncropped western blot for Figure 3H

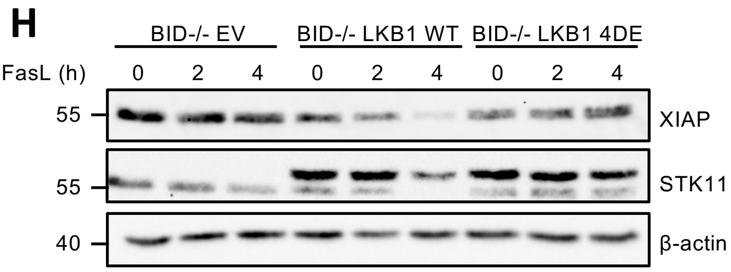

HeLa BID KO #1

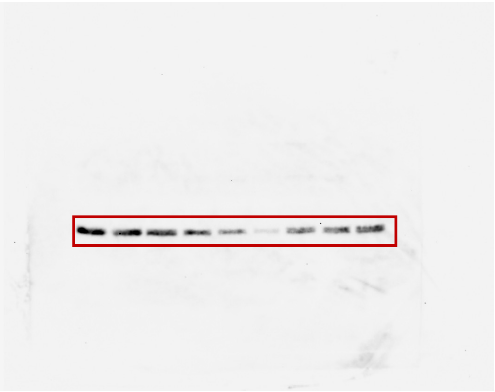

Figure 3H XIAP

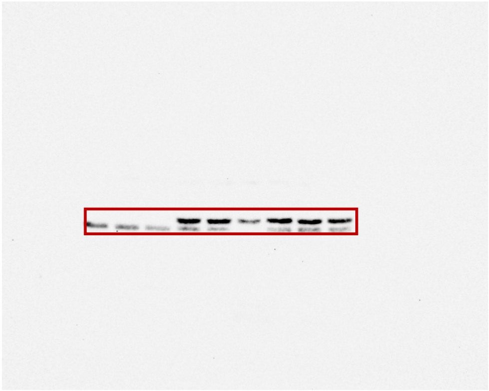

Figure 3H LKB1

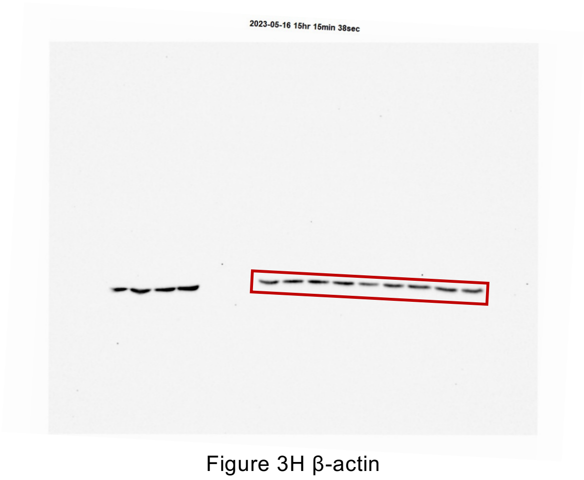

Figure 3H β-actin

Full-length and uncropped western blot for Figure 4B

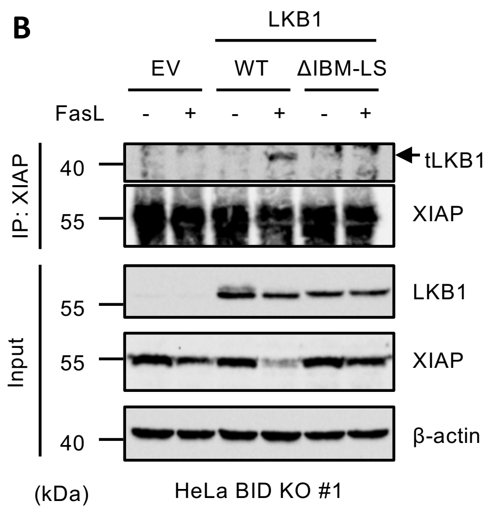

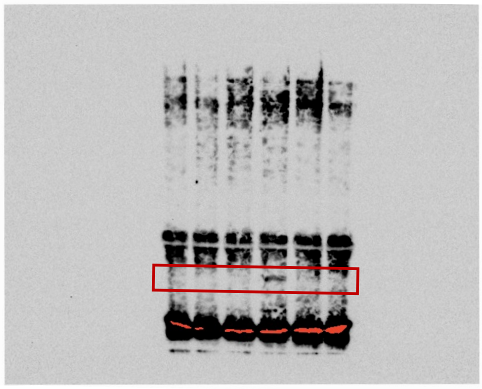

Figure 4B tLKB1 (IP) (IB : LKB1)

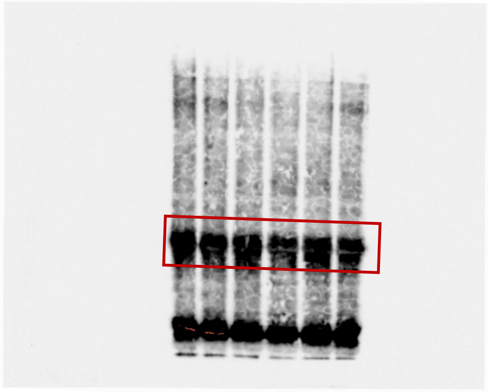

Figure 3F XIAP (IP)

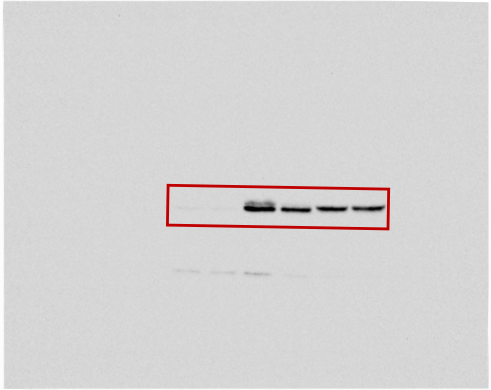

Figure 3F LKB1 (input)

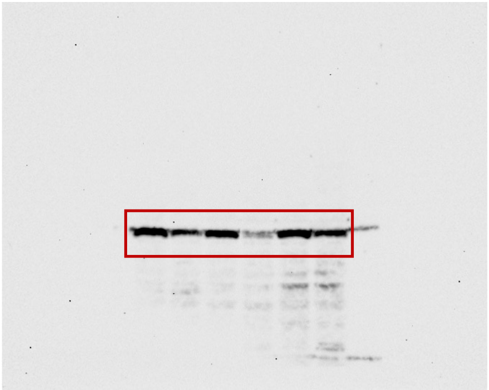

Figure 3F XIAP (input)

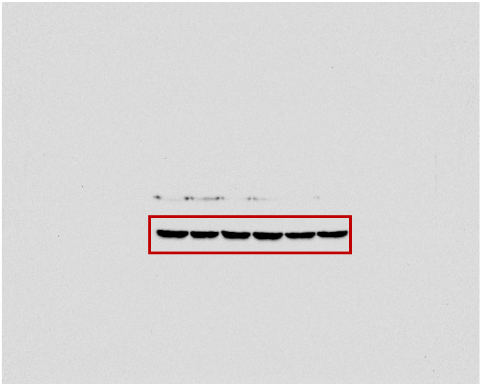

Figure 3F β-actin (input)

Full-length and uncropped western blot for Figure 4C

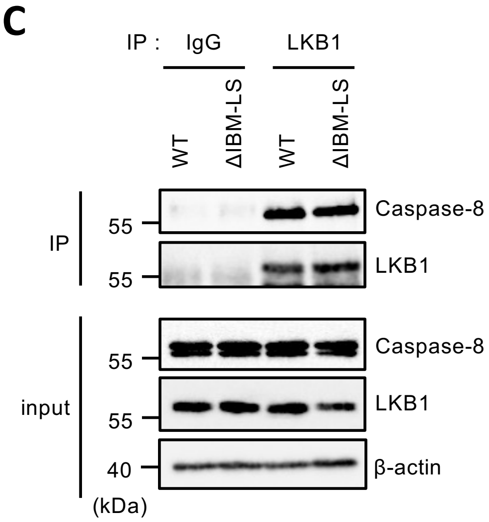

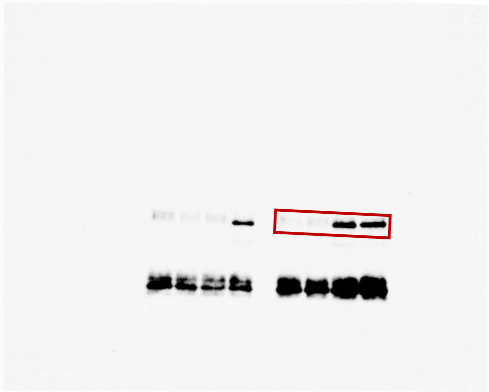

Figure 4C Caspase-8 (IP)

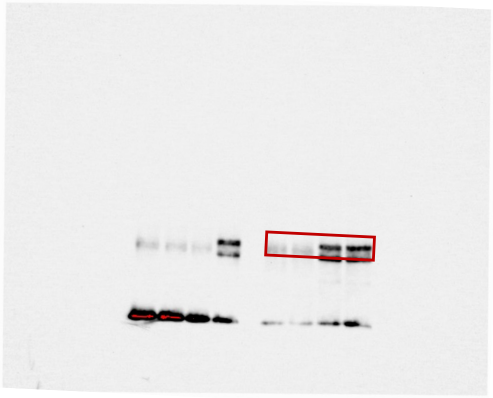

Figure 4C LKB1 (IP)

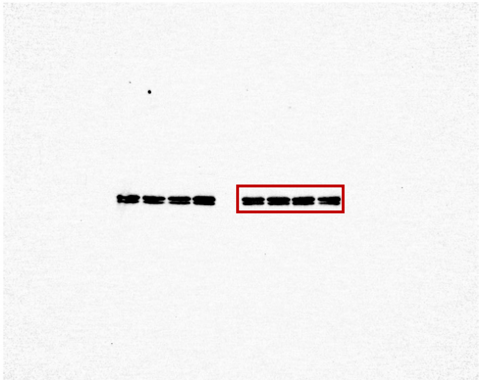

Figure 4C Caspase-8 (input)

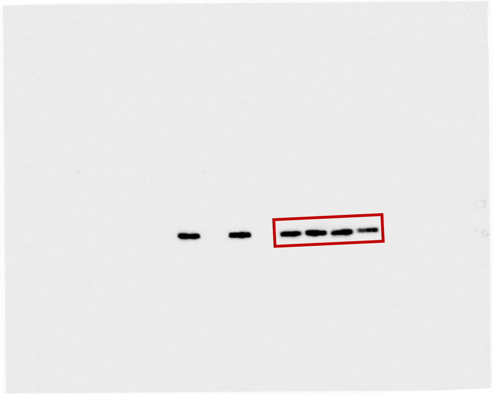

Figure 4C LKB1 (input)

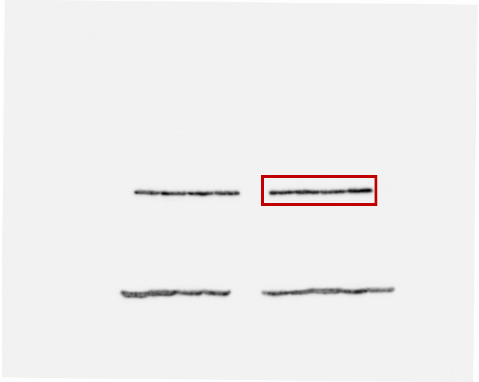

Figure 4C β-actin (input)

Full-length and uncropped western blot for Figure 4D

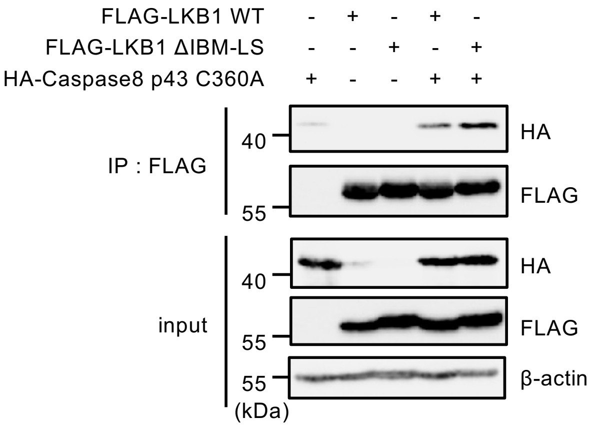

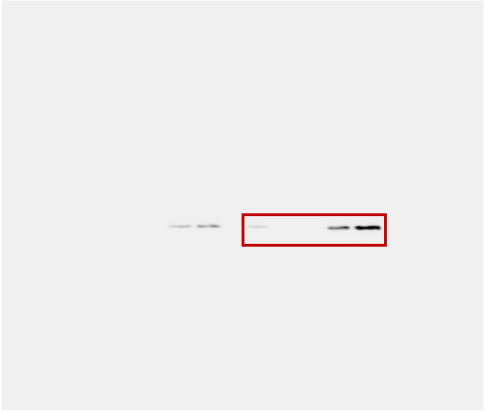

Figure 4D HA (IP)

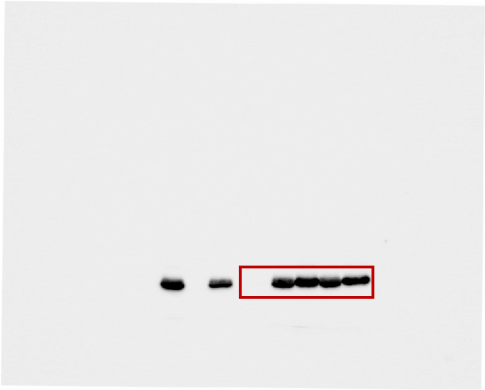

Figure 4D FLAG (IP)

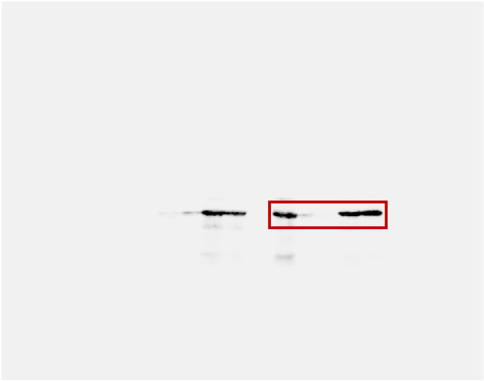

Figure 4D HA (input)

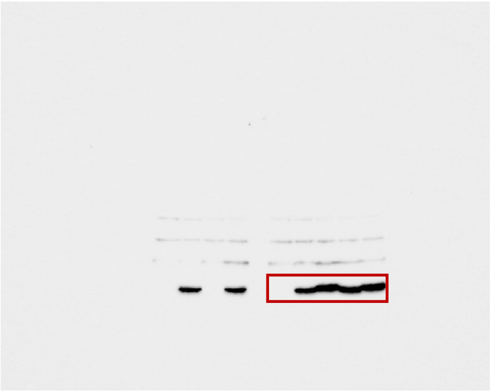

Figure 4D FLAG (input)

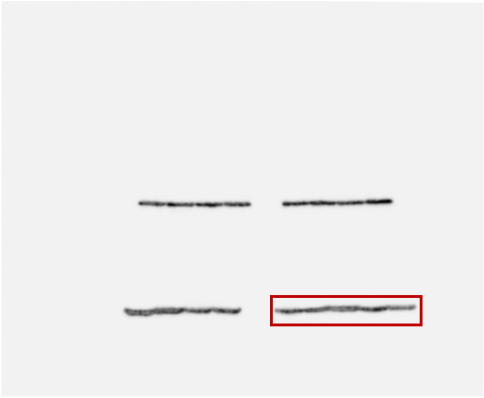

Figure 4D β-actin (input)

Full-length and uncropped western blot for Figure 4E

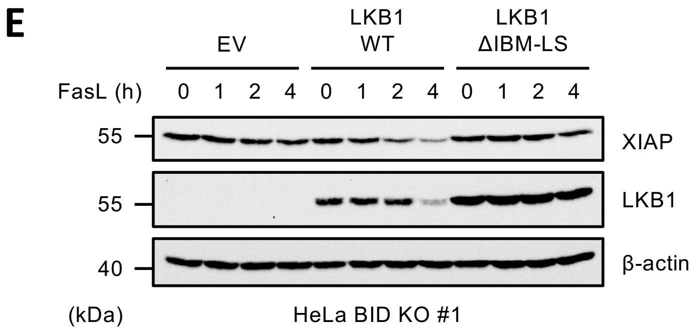

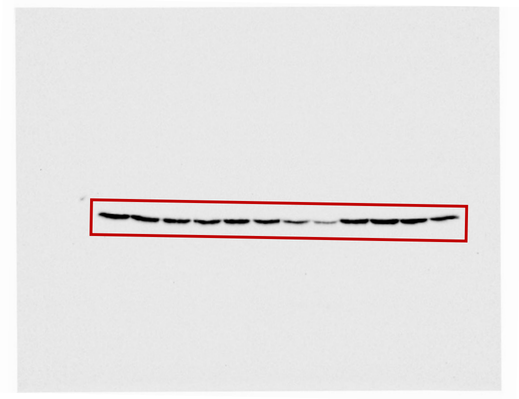

Figure 4E XIAP

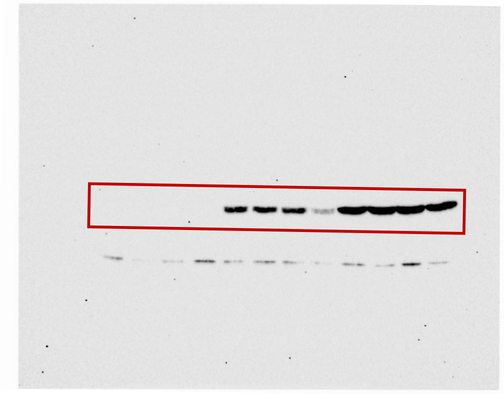

Figure 4E LKB1

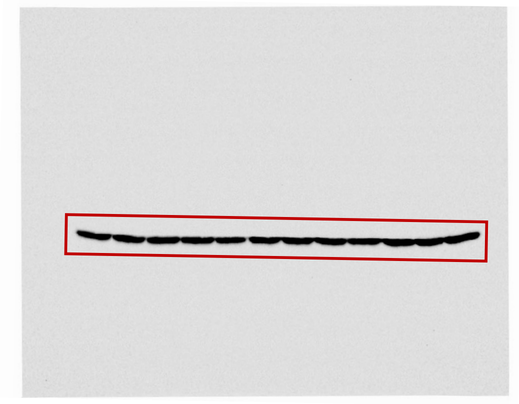

Figure 4E β-actin

Full-length and uncropped western blot for Figure 4F

F

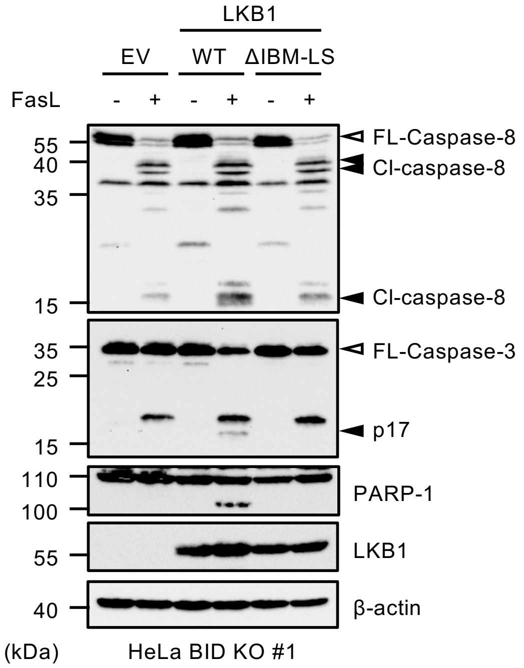

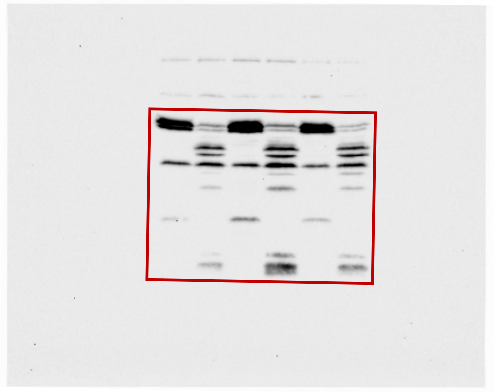

Figure 4F Caspase-8

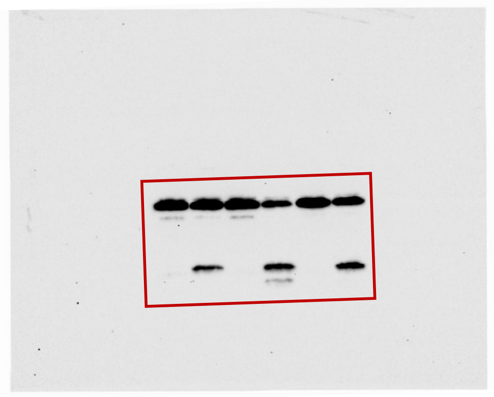

Figure 4F Caspase-3

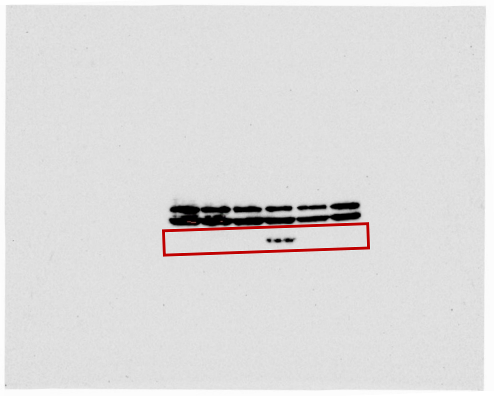

Figure 4F Cleaved PARP (IB : PARP-1)

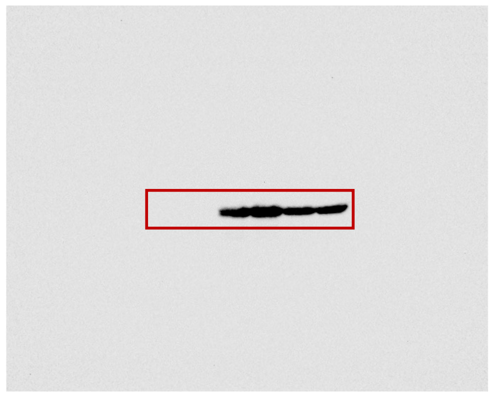

Figure 4F LKB1

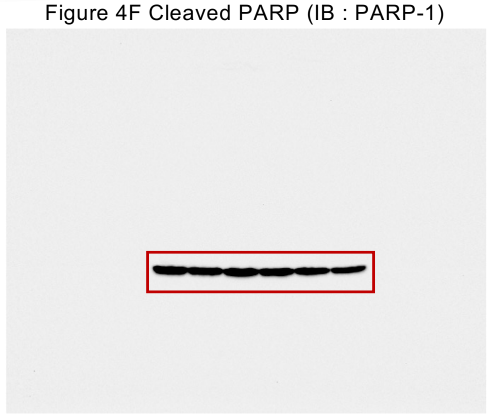

Figure 4F β-actin

Full-length and uncropped western blot for Figure 5A

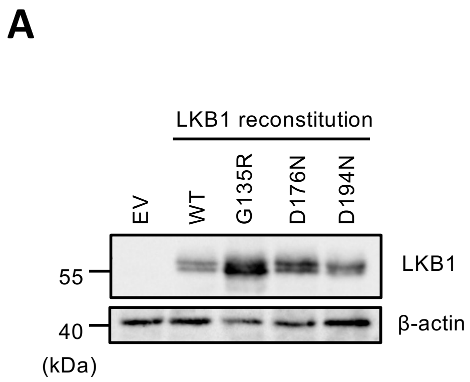

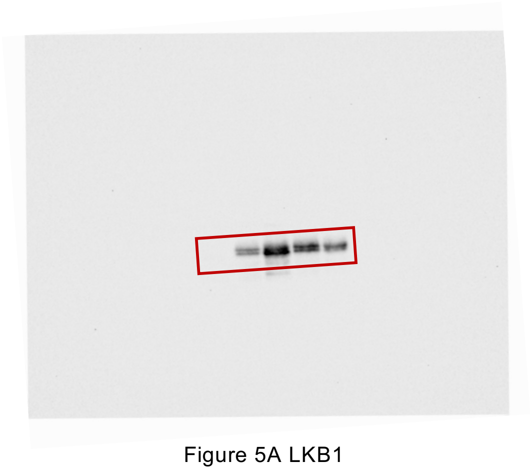

Figure 5A LKB1

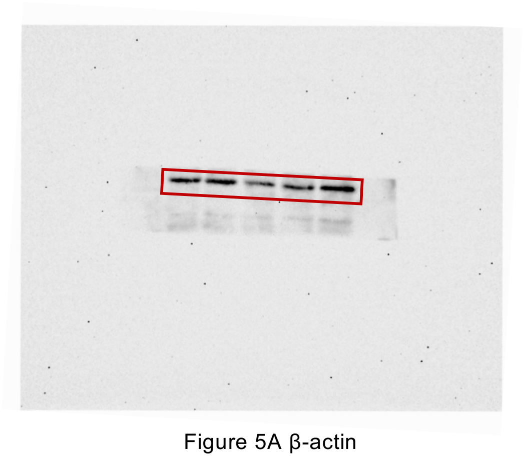

Figure 5A β-actin

Full-length and uncropped western blot for Figure 5B

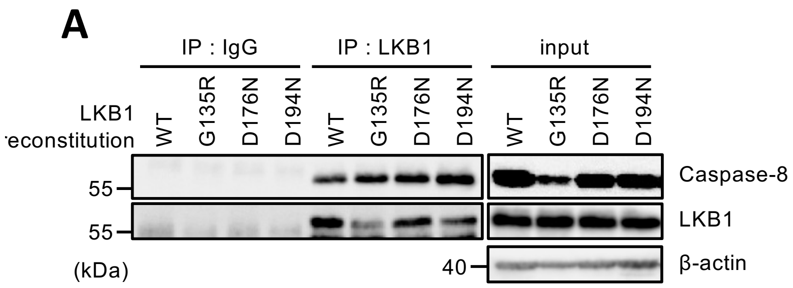

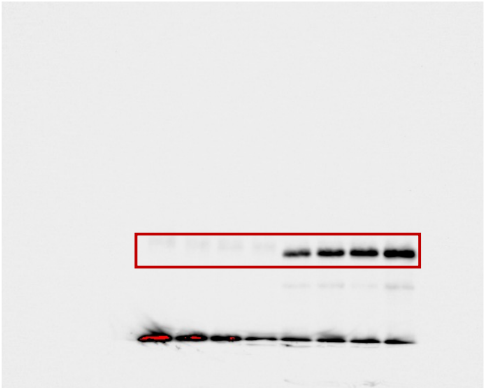

Figure 5B Caspase-8 (IP)

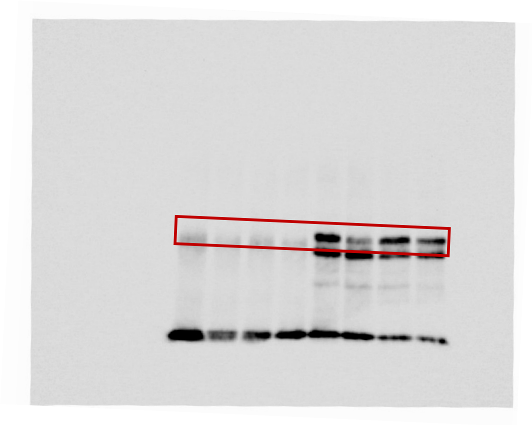

Figure 5B LKB1 (IP)

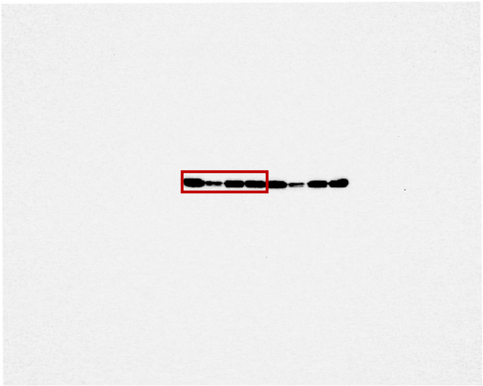

Figure 5B Caspase-8 (input)

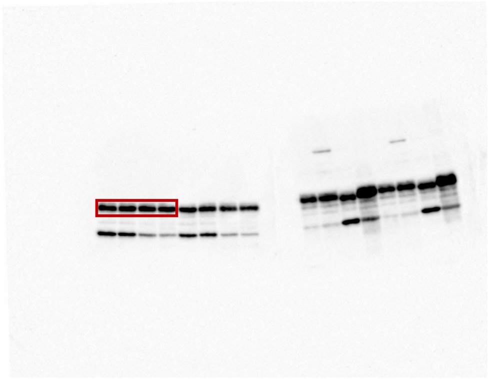

Figure 5B LKB1 (input)

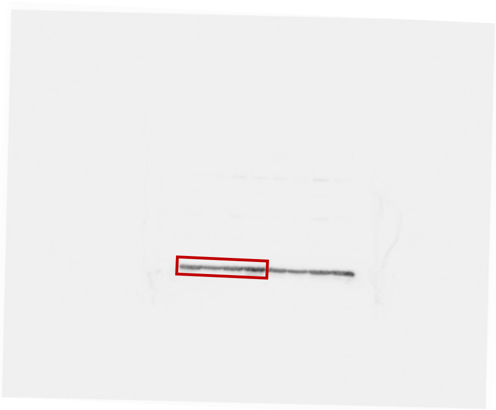

Figure 5B LKB1 (input)

Full-length and uncropped western blot for Figure 5C

C

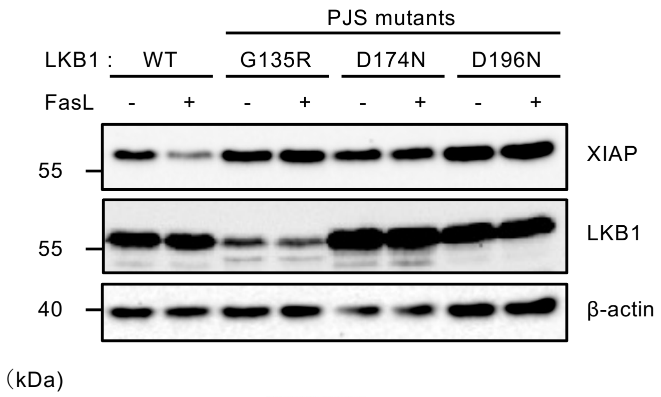

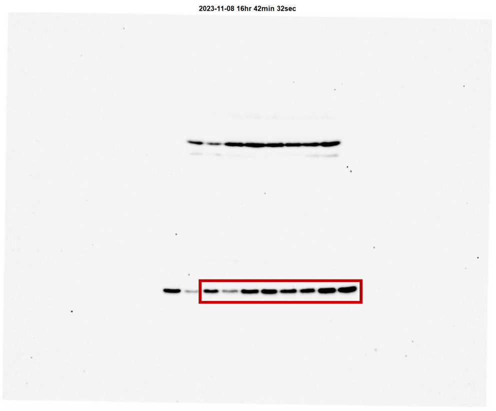

Figure 5C XIAP

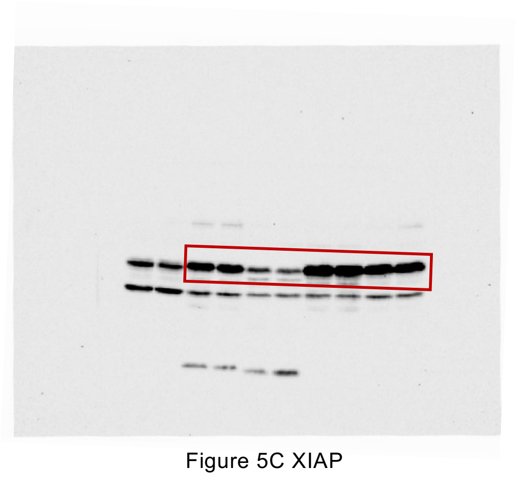

Figure 5C XIAP

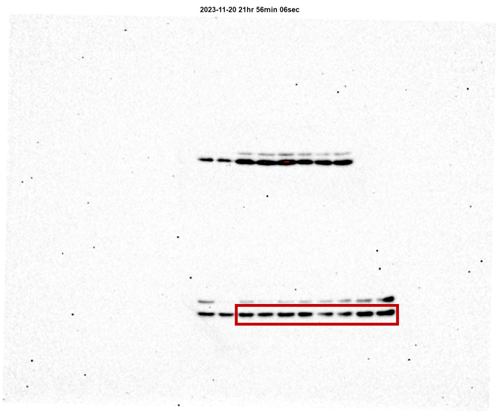

Figure 5C β-actin

Full-length and uncropped western blot for Figure 5D

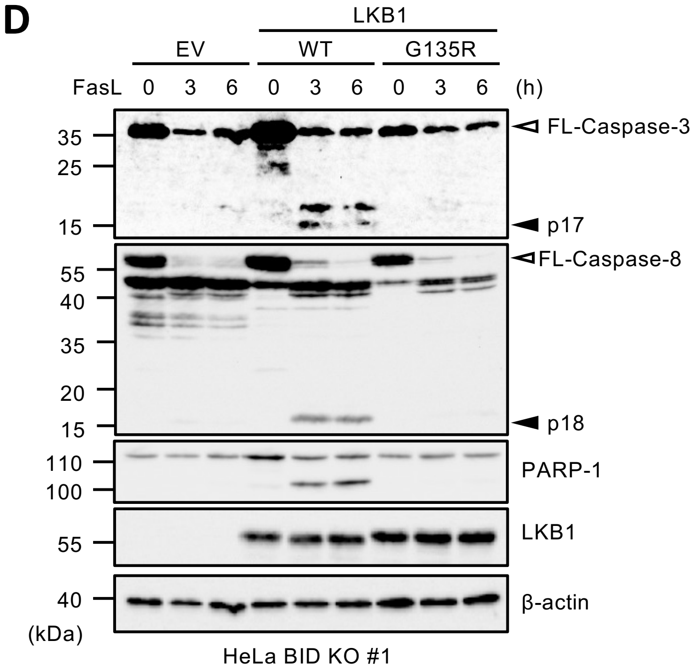

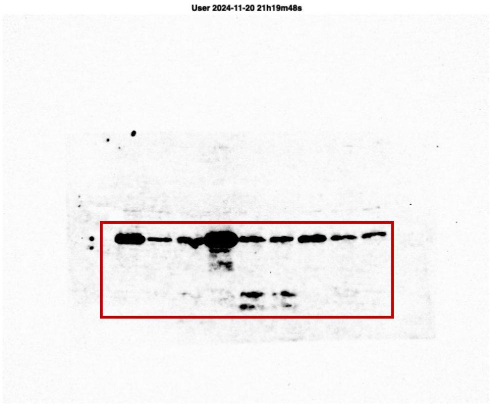

Figure 5D Caspase-3

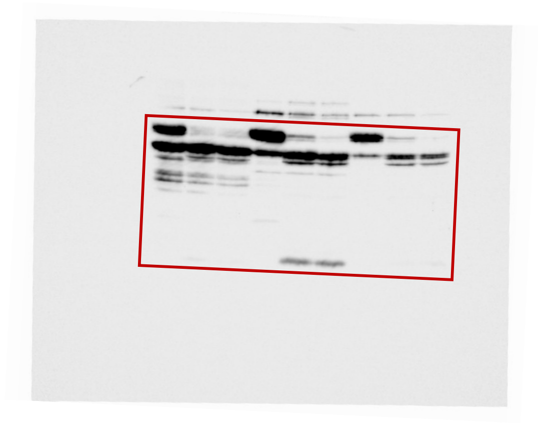

Figure 5D Caspase-8

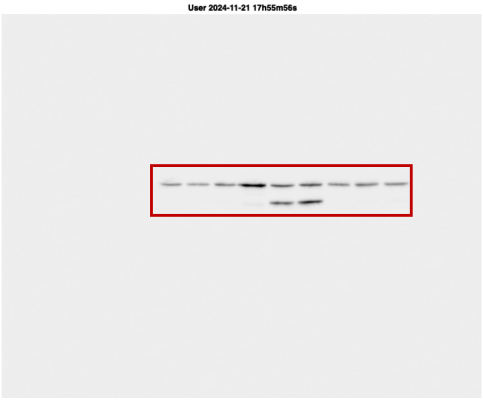

Figure 5D PARP-1

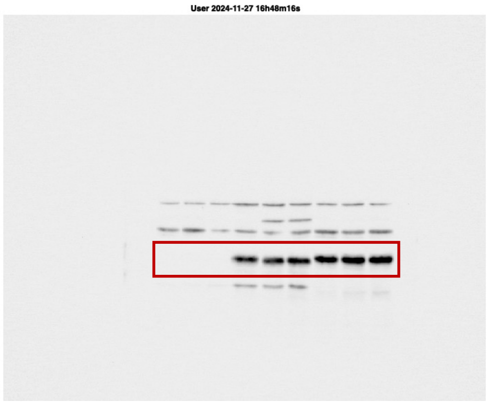

Figure 5D LKB1

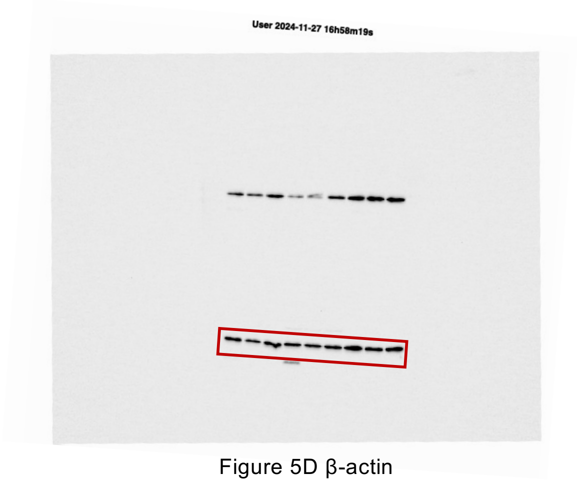

Figure 5D β-actin

Full-length and uncropped western blot for Figure 5E

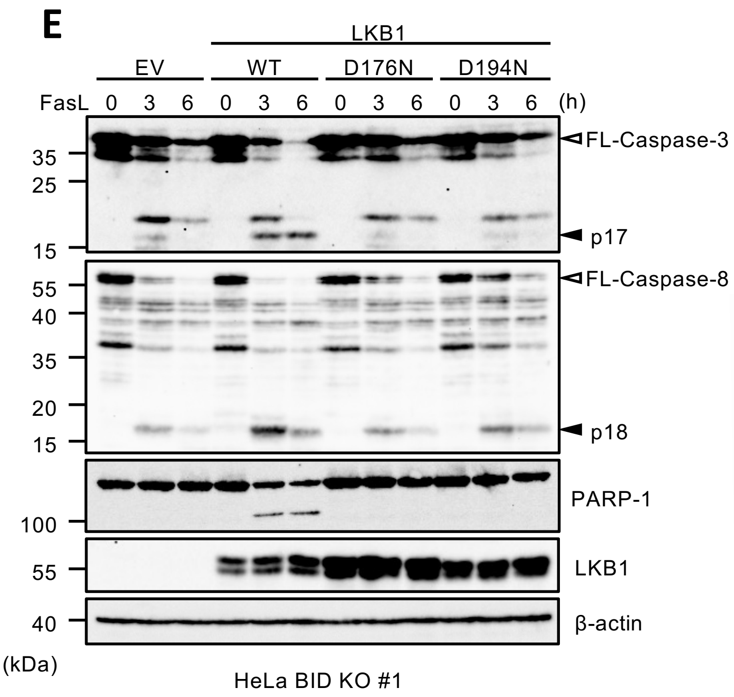

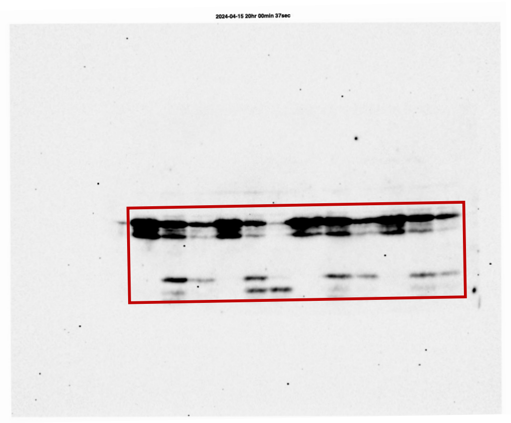

Figure 5E Caspase-3

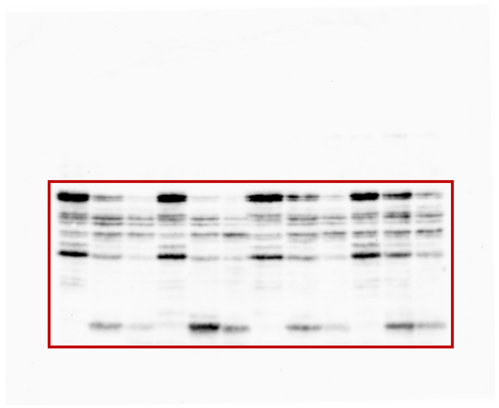

Figure 5E Caspase-8

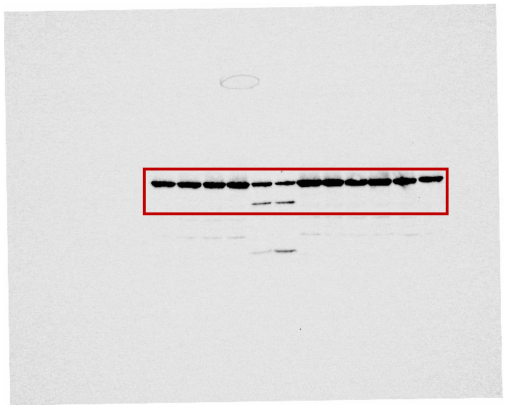

Figure 5E PARP-1

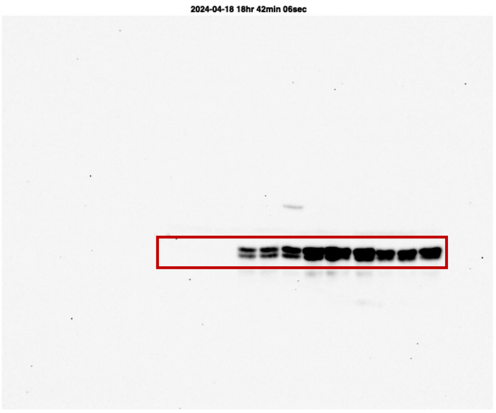

Figure 5E LKB1

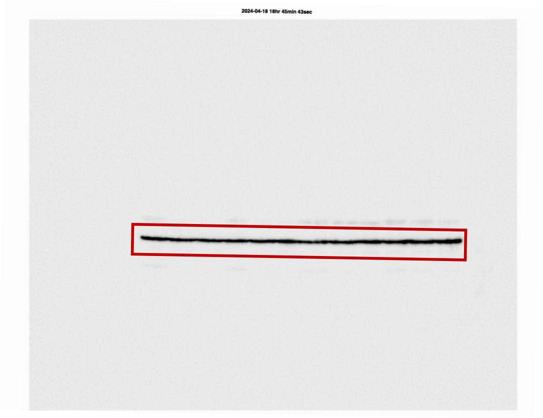

Figure 5E β-actin

Full-length and uncropped western blot for Figure S1F

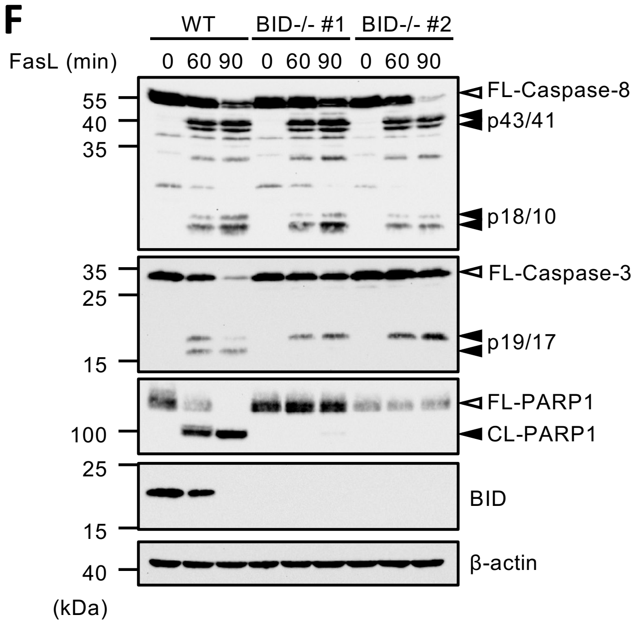

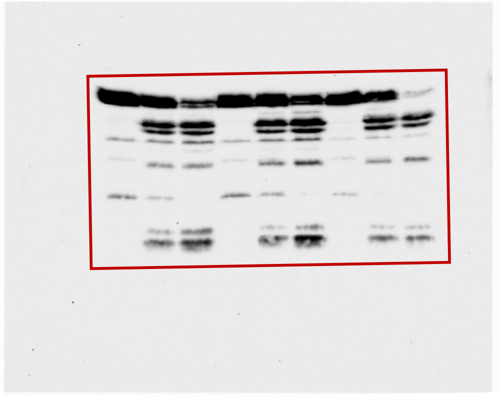

Figure S1F Caspase-8

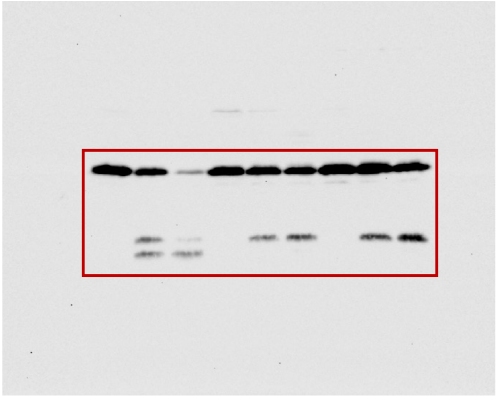

Figure S1F Caspase-3

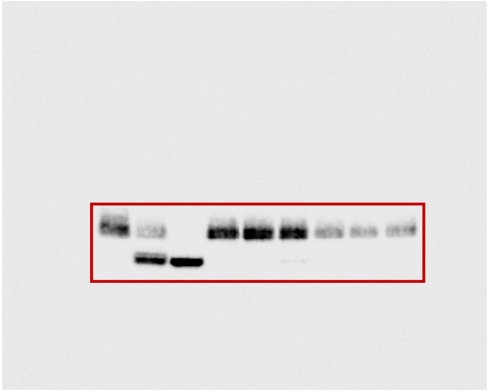

Figure S1F Cleaved-PARP (IB : PARP-1)

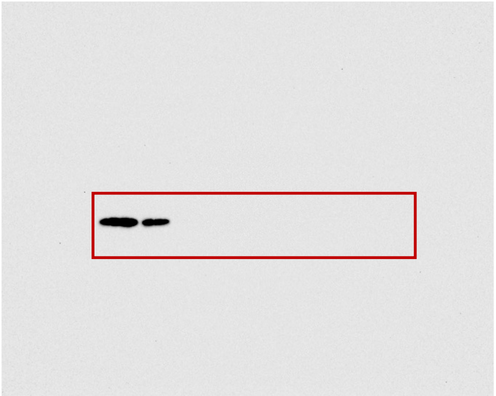

Figure S1F BID

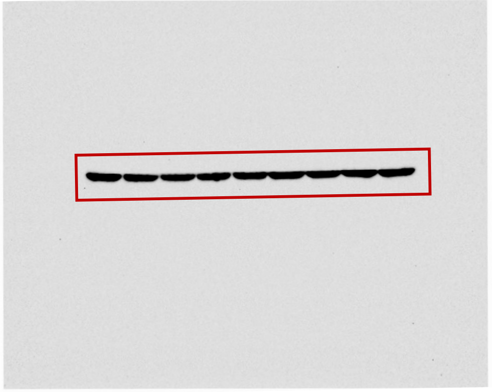

Figure S1F β-actin

Full-length and uncropped western blot for Figure S2A

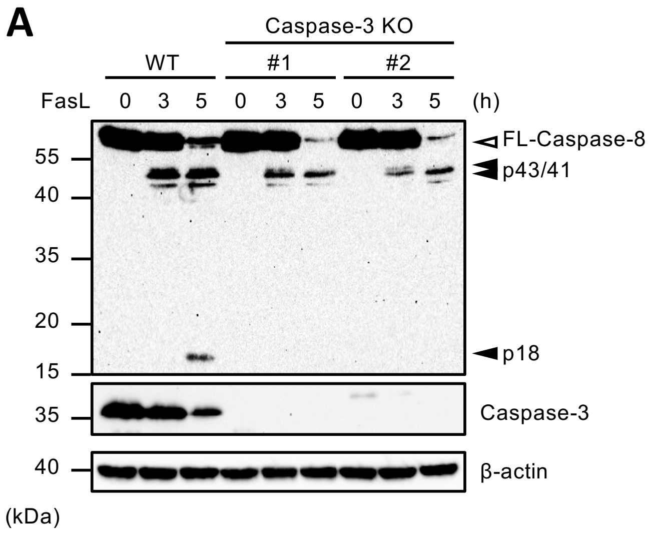

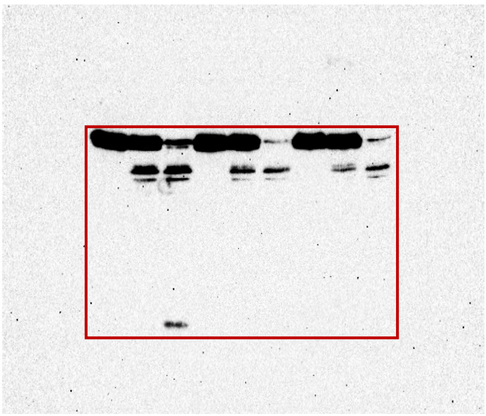

Figure S2A Caspase-8

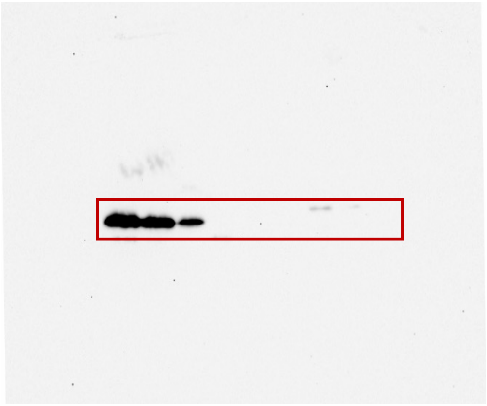

Figure S2A Caspase-3

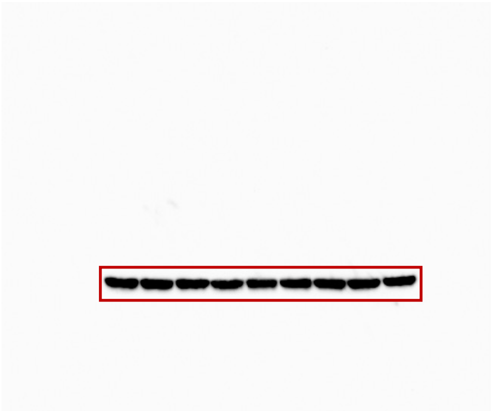

Figure S2A β-actin

Full-length and uncropped western blot for Figure S4A

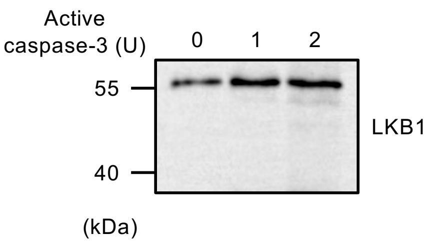

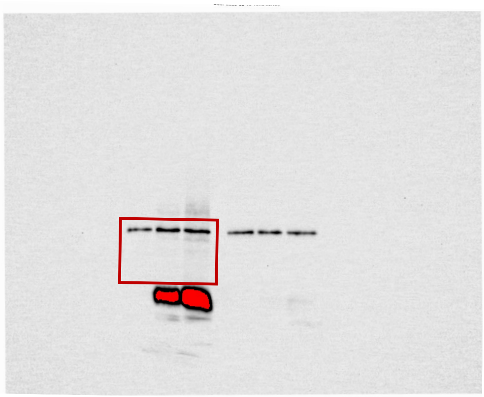

Figure S4A LKB1

Full-length and uncropped western blot for Figure S4A

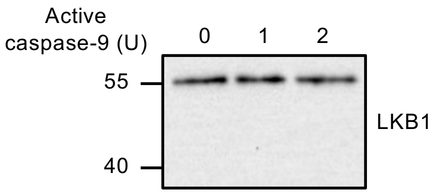

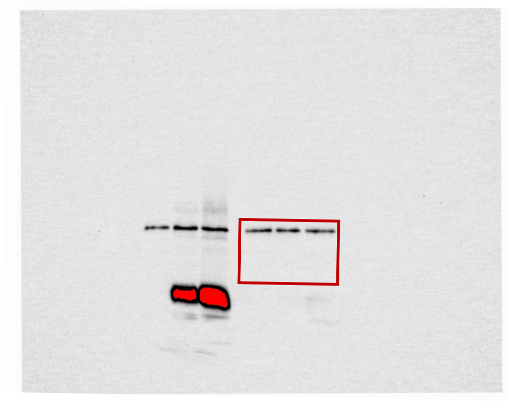

Figure S4B LKB1

Full-length and uncropped western blot for Figure S5A

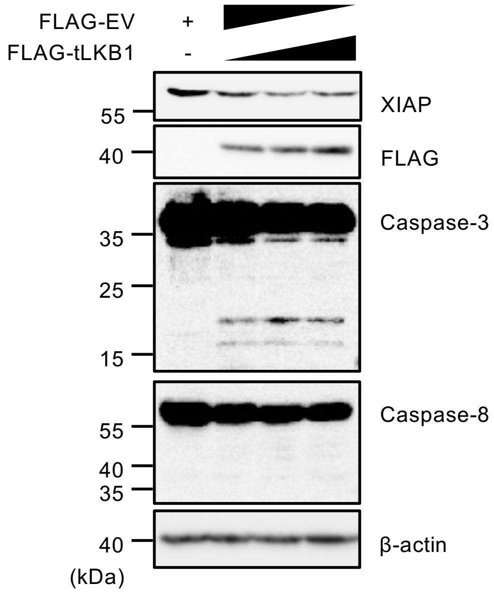

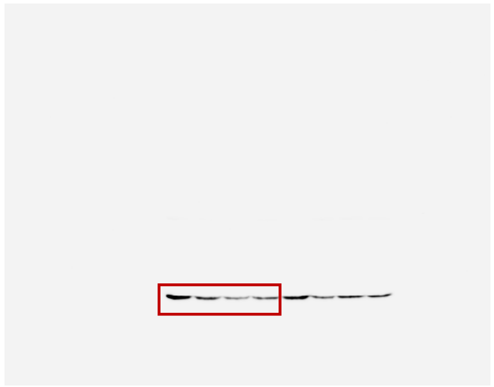

Figure S5A XIAP

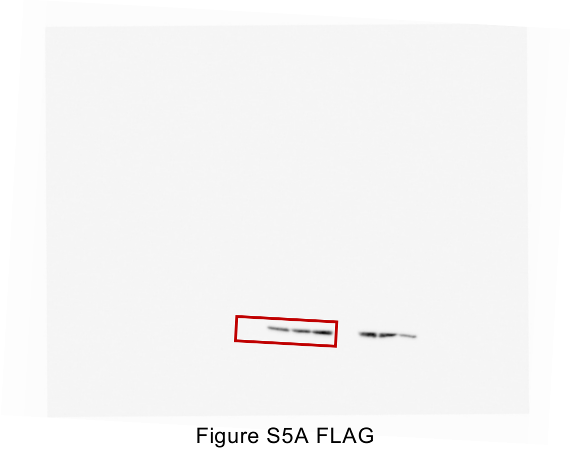

Figure S5A FLAG

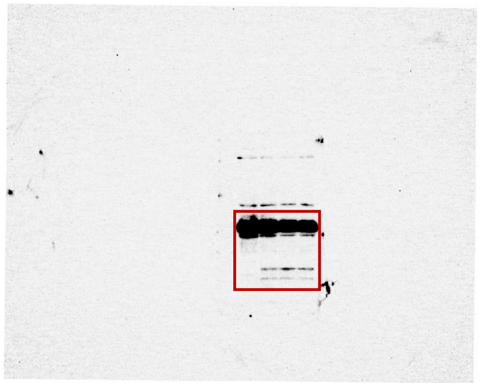

Figure S5A Caspase-3

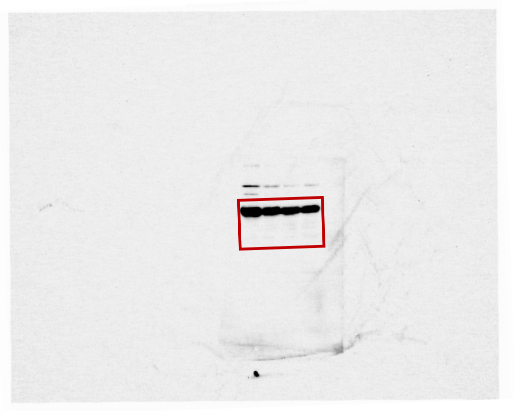

Figure S5A Caspase-8

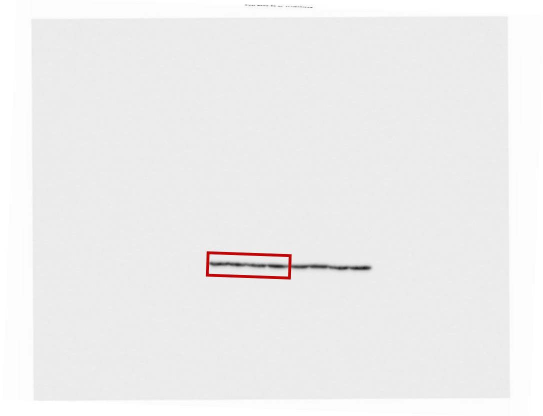

Figure S5A β-actin

Full-length and uncropped western blot for Figure S6A

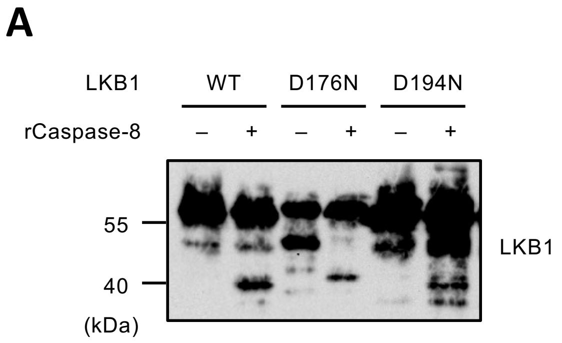

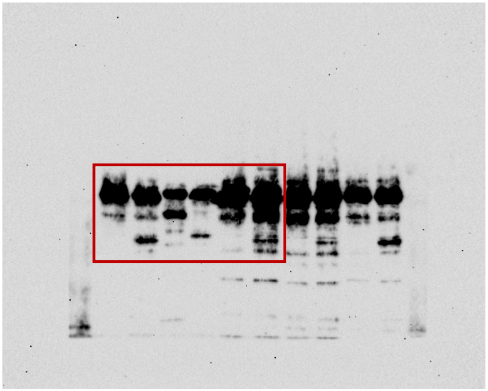

Figure S6A LKB1
